# Functional Characterization of Tea (*Camellia sinensis*) MYB4a Transcription Factor Using an Integrative Approach

**DOI:** 10.3389/fpls.2017.00943

**Published:** 2017-06-12

**Authors:** Mingzhuo Li, Yanzhi Li, Lili Guo, Niandi Gong, Yongzheng Pang, Wenbo Jiang, Yajun Liu, Xiaolan Jiang, Lei Zhao, Yunsheng Wang, De-Yu Xie, Liping Gao, Tao Xia

**Affiliations:** ^1^State Key Laboratory of Tea Plant Biochemistry and Utilization, Anhui Agricultural UniversityHefei, China; ^2^School of Life Science, Anhui Agricultural UniversityHefei, China; ^3^Institute of Botany, Chinese Academy of SciencesBeijing, China; ^4^College of Horticulture, Qingdao Key Laboratory of Genetic Improvement and Breeding in Horticultural Plants, Qingdao Agricultural UniversityQingdao, China; ^5^Department of Plant and Microbial Biology, North Carolina State University, RaleighNC, United States

**Keywords:** tea, *Camellia sinensis*, R2R3-MYB, transcription factor, phenylpropanoids, shikimate pathway

## Abstract

Green tea (*Camellia sinensis, Cs*) abundantly produces a diverse array of phenylpropanoid compounds benefiting human health. To date, the regulation of the phenylpropanoid biosynthesis in tea remains to be investigated. Here, we report a cDNA isolated from leaf tissues, which encodes a R2R3-MYB transcription factor. Amino acid sequence alignment and phylogenetic analysis indicate that it is a member of the MYB4-subgroup and named as CsMYB4a. Transcriptional and metabolic analyses show that the expression profile of *CsMYB4a* is negatively correlated to the accumulation of six flavan-3-ols and other phenolic acids. GFP fusion analysis shows CsMYB4a’s localization in the nucleus. Promoters of five tea phenylpropanoid pathway genes are isolated and characterized to contain four types of AC-elements, which are targets of MYB4 members. Interaction of CsMYB4a and five promoters shows that CsMYB4a decreases all five promoters’ activity. To further characterize its function, *CsMYB4a* is overexpressed in tobacco plants. The resulting transgenic plants show dwarf, shrinking and yellowish leaf, and early senescence phenotypes. A further genome-wide transcriptomic analysis reveals that the expression levels of 20 tobacco genes involved in the shikimate and the phenylpropanoid pathways are significantly downregulated in transgenic tobacco plants. UPLC-MS and HPLC based metabolic profiling reveals significant reduction of total lignin content, rutin, chlorogenic acid, and phenylalanine in *CsMYB4a* transgenic tobacco plants. Promoter sequence analysis of the 20 tobacco genes characterizes four types of AC-elements. Further CsMYB4a-AC element and CsMYB4a-promoter interaction analyses indicate that the negative regulation of CsMYB4a on the shikimate and phenylpropanoid pathways in tobacco is via reducing promoter activity. Taken together, all data indicate that CsMYB4a negatively regulates the phenylpropanoid and shikimate pathways.

**Highlight:** A tea (*Camellia sinensis*) *MYB4a* is characterized to encode a R2R3-MYB transcription factor. It is shown to repressively control the phenylpropanoid and shikimate pathway.

## Introduction

Tea is one of the most popular, non-alcoholic beverages in the world. Numerous studies have reported that tea consumption on a daily basis has beneficial effects on human health and can extend life expectancy (Setiawan et al., 2001; Sueoka et al., 2001). In general, drinking tea can reduce the incidence of cancer (Hertog et al., 1993; Soobrattee et al., 2006), decrease the accumulation body fat (Ogden et al., 2006; Nagao et al., 2007; Auvichayapat et al., 2008), improve glucose homeostasis (Kao et al., 2006; Fukino et al., 2007), and promote cardiovascular health (Nakachi et al., 2000; Ryu et al., 2006). Drinking tea can also prevent against aging-related diseases, such as Alzheimer’s and Parkinson’s diseases (Afzal et al., 2015). Multiple studies have also demonstrated that rich active phenylpropanoid compounds provide nutritional benefits of tea (Sueoka et al., 2001; Liu et al., 2012; Jiang et al., 2013). Phytochemical studies have identified that tea leaves possess abundant flavonoids (such as epicatechin, catechin, oligomeric procyanidins, epicatechin-3-gallate, epigallocatechin-3-gallate, quercetins, and others) and phenolic acids (such as chlorogenic acid, caffeic acid, and others) (Sueoka et al., 2001; Liu et al., 2012; Jiang et al., 2013). All of these metabolites are highly valuable pharmaceuticals.

It is fairly understood that the biosynthesis of phenylpropanoids (e.g., anthocyanins) is regulated by different families of transcription factors such as MYB, bHLH, and WD40 (Zhao et al., 2013). The MYB family is composed of a large number of members, which have been sequenced in numerous different plant genomes. The R2R3-MYB subfamily contains the most members. For example, the genome of *Arabidopsis thaliana* contains 138 R2R3-MYB members (Liu et al., 2015), which are categorized into 25 different subgroups, each characterized by specific regulatory activities. Based on a functional analysis, a few MYBs have been demonstrated to either positively (as activators) or negatively (as repressors) regulate the phenylpropanoid pathway (Dubos et al., 2010; Shi and Xie, 2014; Liu et al., 2015). Examples of well characterized activators include members of the MYB5, MYB6, and MYB7 subgroups, such as Arabidopsis MYB5, MYB75, MYB12, and their homologs in other plants. These activators are specifically involved in the positive regulation of the flavonoid pathway, such as anthocyanin biosynthesis (Xie et al., 2006; Zhao et al., 2013; Shi and Xie, 2014; Zhang et al., 2015; Supplementary Figure S1). A few members of the R2R3-MYB4 subgroup are repressors that have been shown to suppress the biosynthesis of lignin and other phenylpropanoid compounds (Dubos et al., 2010; Supplementary Figure S1). This type of MYB diversity has also been revealed in other plants. For example, the barley genome was recently revealed to transcribe 320 MYB members, which was categorized into different subgroups associating with different regulatory functions (Tombuloglu et al., 2013).

The sequence features of the R2R3-MYB4 subgroup and their regulation activities have been investigated in model plants and a few crops (Tamagnone et al., 1998; Dubos et al., 2010; Fornalé et al., 2010). All characterized members contain the conserved R2R3 domain in the N-terminus. Most members analyzed also contain a C1 (LlsrGIDPxT/SHRxI/L) (Stracke et al., 2001), a C2 (pdLNLD/ELxiG/S), a Zf (CX1–2CX7–12CX2C), and a C4 (FLGLX4–7V/LLD/GF/YR/SX1LEMK) domain in the C-terminus. The C1 domain has a putative activation function (Shen et al., 2012), but, the C2 and C4 domains have been shown to repressively regulate the phenylpropanoid biosynthesis (Matsui et al., 2008; Shen et al., 2012; Zhou et al., 2015). The function of the Zf domain remains largely unknown. To date, multiple R2R3-MYB4 members from herbaceous plant species have been functionally characterized to repressively regulate the phenylpropanoid pathway activity. AmMYB308 and AmMYB330 are two members from *Antirrhinum majus*. The ectopic expression of either of them was found to suppress the biosynthesis of lignin and monolignols (Tamagnone et al., 1998). Four MYB4 members, namely AtMYB3, AtMYB4, AtMYB7, and AtMYB32, have been identified in the genome of *A. thaliana* (Stracke et al., 2001). In *A. thaliana*, AtMYB4 was shown to repress the expression of the *C4H* gene (Jin et al., 2000) and AtMYB7 was demonstrated to repress the expression of *4CL3* and *FLS*, thus leading to the decrease of the biosynthesis of flavonoids (Fornalé et al., 2014). ZmMYB31 and ZmMYB4 are two members in maize. The former was shown to reduce the expression of *COMT* leading to the decrease of caffeic acid. The latter was demonstrated to alter the phenylpropanoid pathway activity in transgenic *A. thaliana* plants and to result in changes in cell wall structure, polysaccharide content, and lignin composition and degradability (Fornalé et al., 2006; Sonbol et al., 2009). TaMYB4 and PvMYB4 are two members that have been isolated from wheat and switchgrass, respectively. The expression of *TaMYB4* represses the transcript levels of *CAD* and *CCR*, which are two genes of the lignin pathway (Ma et al., 2011). A heterogeneous expression of *PvMYB4* in tobacco was reported to result in an abnormal growth of the transgenic plants and a decrease of multiple phenylpropanoids (Shen et al., 2012). Additionally, the *PvMYB4* overexpression in switchgrass increases the efficiency of sugar release from cell wall residues (Shen et al., 2012). The overexpression of the chrysanthemum *CmMYB1* in *A. thaliana* was reported to alter lignin composition and repress flavonoid synthesis (Zhu et al., 2013). In comparison, relatively few MYB4 members have been investigated in the regulation of phenylpropanoids in woody shrubs or trees. To date, one *EgMYB1* gene was cloned from *Eucalyptus* and demonstrated to repress the promoter activity of *CAD2* and *CCR*, two genes involved in the lignin formation (Legay et al., 2007). In addition, two members from gymnosperm plants were recently shown to repressively regulate both phenylpropanoid and shikimate pathways. A PgMYB15 from white spruce [*Picea glauca* (Moench) Voss] was shown to repress the expression of *AroF* (*PgDHS2*) controlling the first step of the shikimate pathway (**Figure 6**) and *Pg4CL* involved in biosynthesis lignin (Bomal et al., 2015). A *Populus* MYB182 was shown to repress the expression of multiple genes involved in the shikimate, anthocyanin, and proanthocyanidin pathways (Yoshida et al., 2015).

Molecular and biochemical studies have been performed to understand the repressive mechanism of the MYB4 subgroup (Legay et al., 2007; Fornalé et al., 2010). Sequence analysis coupled with an Electrophoretic Mobility Shift Assay (EMSA) has identified four types of AC-elements, AC-I (ACCTACC), AC-II (ACCAACC), AC-III (ACCTAAC), and AC-IV (ACCAAAC), which are targets of the MYB4 subgroup members. AtMYB4 was shown to bind AC-I, AC-II, and AC-III (Zhao et al., 2007). PvMYB4 was demonstrated to be able to bind all four types of AC-elements (Shen et al., 2012). EgMYB1was shown to bind the AC-I element alone in the promoter sequence of *EgCAD* (Legay et al., 2007) and ZmMYB31 was shown to bind the AC-I and AC-II elements in the promoter of *ZmCOMT* (Fornalé et al., 2010). These data show the variation of MYB4 members’ binding capacity.

To date, the understanding of MYB4 transcription factors in tea is limited. Our previous transcriptomic studies on leaf and other tissues annotated 73 R2R3-MYB members in tea (Zhao et al., 2013). A homolog of the MYB4 subgroup, namely *CsMYB4a* (derived from CsMYB4-6 annotated previously), was identified among the R2R3-MYB members. In the present study, the full-length sequence of *CsMYB4a* was cloned from young leaves of a commercially important tea variety. Transcriptional and metabolic analyses revealed a negative correlation between its expression level and phenylpropanoid profiles in tea leaves. CsMYB4a repressed the promoter activity of five key genes involved in the phenylpropanoid metabolism in tea plants. The overexpression of *CsMYB4a* led to dwarf, abnormal leaf, and early senescence phenotypes of transgenic tobacco plants. Transcriptomic investigations showed that 20 genes in the shikimate and phenylpropanoid pathways were down-regulated in the *CsMYB4a* transgenic tobacco plants. Metabolic analysis revealed reduction of lignin, phenylpropanoid metabolites and phenylalanine. Sequence analysis was completed to identify four types of AC-elements from the 20 promoters. CsMYB4a-AC element and CsMYB4a-promoter interaction analyses suggested that CsMYB4a could repress the promoting activity of the 20 promoters in tobacco plants. All data indicate that CsMYB4a negatively regulates the phenylpropanoid and shikimate pathways.

## Materials and Methods

### Plant Growth and Sample Collection

We grow more than 2000 *Camellia sinensis var. sinensis cv.* “Longjing 43” plants in the field of the research station at Anhui Agricultural University. These plants are being used for basic and applied research purposes. In this investigation, 15 plants were selected to study *CsMYB4*. Given that green tea industry uses young leaves (**Figure 2A**) developed on new branches in April to produce high quality tea products (Jiang et al., 2013), young leaves of these plants are labeled as the 1st, 2nd, 3rd, and mature (4th) ones from the top (shoot apex) to the bottom of each new branch (**Figure 2A**). We collected the 1st, 2nd, 3rd, and mature leaves separately for different experiments. We also collected young stems, buds (shoot apexes), and roots to perform different experiments. Three biological samples (each from five tea shrubs) were collected for each type of tissue. After collection, all tissues were immediately frozen in liquid nitrogen and subsequently stored at -80°C until late use.

*Nicotiana tabacum* cv. “G28” was used for genetic transformation and *N. benthamiana* was used for subcellular protein localization experiments. Seed germination and plant growth were performed in an environmental chamber provided with a constant temperature of 28 ± 3°C and a 12/12-h (light/dark) photoperiod. The light intensity was set up a 150–200 μmol m^-2^ s^-1^. Any modifications of these conditions are specified within the context.

### Cloning of *CsMYB4a* cDNA and Phylogenetic Analysis

Total RNA was isolated from each frozen tissue using an RNAiso-mate for Plant Tissue Kit (Takara, Dalian, China) and then treated with DNAase to remove any genomic DNA according to the manufacturer’s protocol. The resulting DNA-free RNA sample was used as template for reverse transcription to synthesize the first strand of cDNAs using a PrimeScript^®^ RT Reagent Kit (Takara, Dalian, China) by following the manufacturer’s protocol.

Based on the *CsMYB4a* cDNA sequences that were obtained in our previous study (Zhao et al., 2013), one pair of primers (Supplementary Table S1) was designed to amplify the cDNA of *CsMYB4a* by polymerase chain reaction (PCR) using Phusion HF polymerase (#M0530s, Biolab, New England, Ipswich, MA, United States) according to the manufacturer’s protocol. The thermal gradient program for PCR was: 98°C for 30 s, followed by 30 cycles of 98°C for 10 s, 60°C for 20 s, and 72°C for 30 s, and then a 10 min extension at 72°C. The resulting full length of (735 bp) cDNA of *CsMYB4a* was purified from gel and then cloned into the PMD19-T plasmid (Takara, Dalian, China) to obtain a new plasmid PMD19-T-CsMYB4a for sequencing at BGI^1^.

The open reading frame (ORF) of *CsMYB4a* was deduced to encode 245 amino acids. The resulting amino acid sequence was blasted against proteins curated at NCBI to obtain 16 homologs of the MYB4 subgroup. Amino acid sequences of CsMYB4a and nine homologs were aligned to molecularly characterize function domains using the DNAman software as reported previously (Zhao et al., 2013). In addition, amino acid sequences of CsMYB4a and 17 MYB4 homologs were used to develop a phylogenetic tree using the MEGA6.1 software.

### Construction of a Binary Vector and Tobacco Transformation

The ORF of *CsMYB4a* was cloned into a binary vector using the Gateway^®^ Cloning System (Invitrogen, Carlsbad, CA, United States) as described previously (Lei et al., 2007). Briefly, one pair of primers (Supplementary Table S1) was designed to amplify cDNA for linking to attB adapters. The PCR thermal program was: 98°C for 30 s, followed by 30 cycles of 98°C for 10 s, 60°C for 20 s, and 72°C for 30 s, and then a 10 min extension at 72°C. The amplified cDNA was purified and cloned into the entry vector pDONR207 using the Gateway^®^ BP Clonase^®^ Enzyme mix (Invitrogen, Carlsbad, CA, United States). After sequence confirmation, the entry vector was used to introduce the ORF into the destination binary vector pCB2004 using the Gateway LR Clonase^TM^ enzyme (Invitrogen, Carlsbad, CA, United States). Each cloning step was completed by following the manufacturer’s protocol. The ligation mixture was introduced into *Trans*-T1 competent *E. coli* cells (Takara, Dalian, China), which were selected on agar-solidified LB medium containing 50 ug/ml kanamycin. Ten positive colonies were used for isolation of plasmids and sequencing. The resulting binary expression vector was named pCB2004-Cs*MYB4a* (Supplementary Figure S2), in which Cs*MYB4a* was driven by a 35S promoter and the phosphinothricin gene was used for herbicide selection. This new binary vector was subsequently introduced into *Agrobacterium tumefaciens* strain EHA105 by electroporation. A positive EHA105 colony was selected on agar-solidified medium containing 50 mg/L kanamycin and 50 mg/L spectinomycin for genetic transformation of tobacco plants by following a reported leaf disk protocol (Maiti et al., 1988). Potential transgenic plants were selected on agar-solidified MS medium supplemented with 25 mg/L of phosphinothricin. Putative transgenic plants were confirmed using both genomic DNA based PCR and RT-PCR. Positive transgenic plants were grown on soil and placed in an environmental chamber for flowering and seeding. Phenotypes of transgenic and wild-type control plants were observed daily and recorded to compare plant growth.

### Subcellular Localization

The ORF of *CsMYB4a* in the entry vector *CsMYB4a*-pDONR207 was cloned to the destination binary vector, namely pGWB5, for subcellular localization. The cloning procedures were completed by following the Gateway cloning method and the LR clonase reaction as describe above. A positive vector was successfully obtained and named as pGWB5-*CsMYB4a*, (Supplementary Figure S2), in which the ORF was fused at the N-terminus of an EGFP. As described above, this plasmid was introduced into *A. tumefaciens* strain EHA105 to select a positive colony for infiltration of *N. benthamiana* according to a reported protocol (Shen et al., 2012). After 48 hrs of infection, leaves were examined using an Olympus FV1000 confocal microscope (Olympus, Tokyo, Japan). EGFP fluorescence was digitally recorded and analyzed to characterize the subcellular localization of CsMYB4a.

### Promoter Sequence Mining of Twenty *Nicotiana tabacum* Genes and Isolation of Five *Camellia sinensis* Promoters

Promoter sequences of 20 genes involved in the phenylpropanoid and shikimate pathways were identified from the genomic sequences of tobacco^2^. The 20 genes and their corresponding GenBank ID# were *AROF* (NM_001325203.1), *AROB* (XM_009614286.2), *AROK* (XM_009627657.2), *AROA2* (XM_009782781.1), *AROC* (XM_009784811.1), *CM* (XM_009615669.2), *PAL1* (NM_001325423.1), *PAL2* (EU883670.1), *C4H* (NM_001325516.1), *4CL1* (NM_001325625.1), *4CL2* (NM_001325738.1), *CCR* (XM_016587861.1), *CCOMT5* (XM_016619959.1), *CCOMT6* (NM_001325865.1), CAD1 (NM_001325471.1), *COMT* (XM_016634241.1), *CHS-G-like* (XM_016660053.1), *DFR* (NM_001325732.1), *FLS* (XM_016611945.1), and *UFGT* (AB072919.1). The full names of these genes are included in supplementary data (Supplementary Table S6). Based on the localization of these 20 genes in the tobacco genome sequence, we identified 1.6 kb nucleotides located at the immediate upstream of each coding region. We copied all 20 promoter sequences, pasted as fasta format in text sheet, and then analyzed them one by one to identify AC-element using “Promoter analysis” function in the PlantPan^3^ (Chang et al., 2008).

*CsC4H, Cs4CL, CsCHS, CsLAR*, and *CsANR* were selected to isolate their promoter sequences from tea. Primer pairs (Supplementary Table S1) were designed to amplify 1 kb nucleotides from the immediate upstream of these gene coding regions. The Genome-walker kit (Takara, Dalian, China) (Vineet Kumar et al., 2014) was used to isolate these promoter fragments according to the manufacturer’s protocol. The resulting DNA fragments were cloned into the PMD19-T plasmid (Takara, Dalian, China) by following the manufacturer’s protocol. Sequencing was completed at BGI. Sequence analysis and AC-element characterization were carried out at the PlantPan as described above (Chang et al., 2008).

### Electrophoretic Mobility Shift Assay (EMSA)

Electrophoretic mobility shift assay was carried out to understand whether CsMYB4a could bind the four types of AC-elements. This assay was completed with three steps, obtainment of recombinant CsMYB4a, synthesis of AC-element probes, and binding. Firstly, the ORF of *CsMYB4a* was cloned into the *PRSF* protein expression vector, which included a *BamH*I and *Sacl*I restriction site (Jeon et al., 2009). To perform this cloning, one pair of primers was designed to amply the ORF. The forward and reverse primers were added a *BamH*I and *Sacl*I restriction site, respectively. PCR was completed using the PMD19-T-CsMYB4a plasmid as template and a thermal program that was composed of 98°C for 30 s, followed by 30 cycles of 98°C for 10 s, 60°C for 20 s, and 72°C for 30 s, and then a 10 min extension at 72°C. Subsequently, both of the PCR products and the PRSF plasmid were digested with *BamHI* and *SaclI*. Ligation reaction was carried out using an *NEB* T4 ligase according to the manufacturer’s protocol (NEB, Ipswich, MA, United States). The ligated products were transferred to competent BL21 (DE3) *E. coli* cells (Takara, Dalian, China) according to the manufacturer’s protocol. On an agar-solidified LB medium supplemented with 0.5 mM kanamycin, three positive colonies were obtained to isolate plasmids for sequence analysis. One positive colony was then selected and cultured to induce protein expression using 0.5 mM isopropyl β-D-1-thiogalactopyranoside (IPTG) as previously reported (Jeon et al., 2009). The resulting recombinant CsMYB4a protein was purified using a His-tag Protein Purification kit (Clontech, Dalian, China) and stored at -80°C until EMSA assay. Secondly, oligomeric nucleotides (Supplementary Table S1) were designed for synthesis to develop AC-I, II, III, and IV element probes. Four oligomers were synthesized at Shenshi Life (Shanghai China). Finally, the binding experiments were performed according to a protocol (Shen et al., 2012).

### Dual Luciferase Assay

Dual luciferase assay was performed via three steps, development of reporter constructs, selection and development of plasmid expressing CsMYB4a, and interaction of CsMYB4a and promoters. Firstly, seven constructs each containing a promoter sequence were developed for this dual luciferase assay. Primer pairs (Supplementary Table S1) were designed to amplify those promoter sequences of five tea genes (*CsC4H, Cs4CL, CsCHS, CsLAR*, and *CsANR2*) and of two tobacco genes (*AROF* and *AROC*). As described above, five plasmids were developed, each containing one tea gene promoter sequence. Each plasmid was used as template to amplify each gene promoter. The thermal program was composed of 98°C for 30 s followed by 30 cycles of 98°C for 10 s, 60°C for 20 s, and 72°C for 30 s, and then a 10 min extension at 72°C. For amplification of these two tobacco gene promoters, tobacco genomic DNA was isolated from leaf tissue according to a reported protocol (Hou et al., 2016). The thermal program was composed of 98°C for 30 s followed by 30 cycles of 98°C for 10 s, 60°C for 30 s, and 72°C for 30 s, and then a 10 min extension at 72°C. The length of amplified DNA fragment was 1 kb for all five tea genes and 1.6 kb for two tobacco genes. Each promoter was cloned into the PGWL7 plasmid (Supplementary Figure S2) to develop seven new promoter analysis vectors; PGWL7-pC4H, PGWL7-p4CL, PGWL7-pCHS, PGWL7-pLAR, PGWL7-pANR, PGWL7-pAROF, and PGWL7-pAROC, which was subsequently used for dual luciferase assay as reported (Shen et al., 2012).

Secondly, the ORF of *CsMYB4a* was cloned into the destination vector PCB2004 using the Gateway cloning system according to a protocol reported previously (Lei et al., 2007). The resulting new plasmid was named as PCB2004-CSMYB4a, which was used to perform interaction assay between CsMYB4a and each of eight promoters.

Thirdly, the binding of CsMYB4a to promoters was carried out according to an established method (Shen et al., 2012). In addition to PCB2004-CSMYB4a, the PCB2004 empty vector was used as a control. In brief, an *Arabidopsis* protoplast culture was developed from leaf tissues according to a reported protocol (Asai et al., 2002) with minor modifications. A digestion buffer was prepared using 225 mg of cellulose and 45 mg of macroenzyme, which were dissolved in 15 ml of double distilled water and then sterilized by filtration through a 0.2 μm membrane. Young leaves from 30-day-old *Arabidopsis* plants were cut into 0.5–1 mm long pieces. All leaf disks were then completely submerged in the digestion buffer contained within petri dishes. After 3 h of digestion in the dark at room temperature, protoplasts were collected for transformation. Based on a PEG-mediated method (Kyoko et al., 2008), promoter vectors (10 μg) and PCB2004-CsMYB4a (10 μg) or PCB2004 (control plasmid, 10 μg) were added to the protoplast (100 ul,10^5^ protoplasts/ml) mixture within petri dishes, and gently mixed for 30 min. Treated protoplasts were cultured for 6–8 h in the dark at room temperature. Protoplasts were examined under a fluorescence microscope and firefly luciferase/renilla luciferase fluorescence excitation was recorded at a 580 nm wavelength. Vmyb and Ve (Value of CsMYB4a and empty vector) were used as two symbols to represent the excitation values obtained from protoplasts transformed by PCB2004-CSMYB4a and the promoter vectors and by the empty vector control and promoter vectors, respectively. The quotient of Vmyb/Ve was calculated to evaluate each promoter activity. In this assay, three independent repetitive experiments were completed for protoplast isolation and dual-luciferase analysis.

### Transcriptomics and Data Mining

Total RNA was isolated from three biological samples of 30-day-old seedlings of wild-type and transgenic tobacco plants (line 4) (Supplementary Figure S3B) using the Plant Tissue Kit (Takara, Dalian, China) according to the manufacturer’s protocol. RNA quality was evaluated on an Agilent 2100 Bioanalyzer and then used to construct cDNA libraries for next generation sequencing at BGI (BGI, Shenzhen, China). cDNA libraries were constructed according to a previously described method (Tai et al., 2015) and sequencing was conducted using the Illumina HiSeq^TM^ 2000 platform. According to an established pipeline (Tai et al., 2015), all reads were assembled to contigs. All sequence data have been submitted to the GenBank and the accession number is SRR5341129. FPKM values for all of the annotated cDNAs were calculated in order to compare their transcriptional levels in transgenic vs. wild-type plants.

Data mining and identification of unigenes were performed using the BLASTX software (Cameron et al., 2004). Biosynthetic pathway analyses were completed using the KEGG database. Sequences of cDNAs encoding enzymes involved in the phenylpropanoid pathway and shikimate pathway were specifically examined in order to characterize their transcriptional level according to Kanehisa et al. (2008). These genes were then uploaded to the KEGG database to map biosynthetic pathways.

### Quantitative Reverse Transcription-PCR Analysis

Quantitative RT-PCR analysis was performed using SYBR-Green PCR Mastermix (Invitrogen, Carlsbad, CA, United States) on a CFX96^TM^ (Bio-RAD, California, CA, United States). Gene-specific primer pairs and different thermal programs (Supplementary Table S1) were developed to compare expression levels of 20 genes in transgenic vs. wild-type plants. These genes were putative targets of MYB4a as described in transcriptomics analysis above. *ACTIN* was used as reference control for normalization. Amplified products were monitored using an optical reaction module and the resulting amplified values of genes were normalized against those of *ACTIN* according to a protocol for tobacco plants (Pang et al., 2007). Steps of PCR analysis were carried out as described previously in our laboratory (Zhao et al., 2013). Three technical replicates were completed in this assay for each gene expression analysis.

### Analysis of Lignin in Transgenic and Wild-Type Tobacco Plants

Lignin analysis for three transgenic lines (line2, line4 and line5) and wild-type cotrol plants was carried out using a reported Syros method (Halpin et al., 1994) with minor modifications. Thirty-day old seedlings (including roots, stems, and leaves) were collected and then were ground into fine powder in liquid nitrogen. One hundred mg of powder was extracted with 1 ml of 50% ethyl alcohol (in water) in a 2 ml tube at 80°C. After 3 h of extraction, 1 ml methanol was added to the mixture, which was then incubated for additional 1 h at 80°C. Treated samples were centrifuged to obtain supernatant and residue phases. The supernatant was then discarded and the remaining residue was subsequently dried to powder at 60°C in an oven. Ten milligrams of dry residue powder was extracted for 30 min in 5 ml 25% (w/w) acetyl bromide in acetic acid in a 1.5 ml tube. The extract was treated for 30 min with 0.2 ml 70% perchloric acid in water at 70°C. After cooling to room temperature, tubes were centrifuged at 3000 × *g* for 15 min to separate the supernatant from the residue. The supernatant was pipetted into a new 50 ml tube. Five ml of 2 M NaOH was added to each tube. The volume of the mixture was increased to 25 ml with glacial acetic acid and then mixed well. One ml of the mixture was used to measure absorbance at 280 nm in a UV-visible Hitachi U-5100 spectrophotometer (Hitachi, Tokyo, Japan). Absorbance values were used to estimate lignin contents according to a method reported previously (Piquemal et al., 2002). Three biological replicates were completed for transgenic and wild-type plants. The mean value of three replicates was calculated and then statistically evaluated using the SPSS software to compare lignin contents between transgenic vs. wild-type plants.

Microscopic observation of plant tissues was also conducted to visualize alterations of lignin deposition in the tissues of transgenic plants. The basal stems of 30-day old tobacco seedlings were crossly sectioned to 30 μm thick slices using a freezing microtome. Frozen slices were immediately placed in 1% phloroglucinol (dissolved in 92% ethanol) for 2 min, followed by the addition of 25% HCl for 3 min to colorize lignin. Cross sections were removed from the lignin stain reaction and rinsed 5–6 times using double deionized water to remove traces of the dye solutions. Cross sections were then mounted on glass slides and observed under a light microscope and photographed to show the pattern and staining level of lignin.

### Extraction of Flavan-3-ols and Phenolic Acids from Tea Leaves

Phenolic compounds were extracted using the following procedure. Fresh leaves and buds from five positions of new branches (**Figure 2A**) were collected to liquid nitrogen. In addition, woody roots and stems were collected for analysis. Samples were ground into fine powder in liquid nitrogen. Powdered samples (150 mg) were suspended in 1 ml 80% methanol: 1% hydrochloric acid in a 1.5 ml tube. Tubes were completely vortexed and then placed in the room temperature for 20 min, followed by 10 min of centrifugation at 12,000 × *g*. The supernatant was transferred to a new tube. This extraction was repeated once to obtain a final volume of 2 ml extract. All extracts were then filtered through a 0.22 μm membrane into a new tube prior to HPLC or LC-MS analysis described below. Three biological replicates were completed for each tissue.

### Extraction of Flavonoids, Phenolic Acid, and Phenylalanine from Transgenic vs. Wild-Type Tobacco Plants

Thirty-day old tobacco seedlings, both transgenic and wild-type, were collected as described above for extraction of tea flavan-3-ols and phenolic acid. Three biological replicates were completed for transgenic and wild-type samples.

Phenylalanine extraction was carried out based on a previously published protocol (Zhang et al., 2010). In brief, lyophilized tobacco seedling tissue (0.15 g) was extracted twice in 4 ml of boiling water for 15 min. The resulting supernatants were collected and then adjusted to a volume of 10 ml with water. A 10 μl sample was mixed with 70 μl of borax buffer (pH 8.0) and 20 μL of ACCQ (6-aminoquinolyl-*N*-hydroxyl-succinimidyl carbamate) fluor derivation reagent and incubated at 55°C for 20 min prior to HPLC analysis.

### Analysis of Metabolites by UPLC-MS/MS and HPLC

Flavan-3-ols, phenolic acids, and other flavonoids were analyzed using ultra-high performance liquid chromatography (UPLC)-MS/MS on an Agilent LC-MS system (Palo Alto, CA, United States). Metabolites were separated in an Agilent 20RBAX RRHD Eclipse Plus C18 column (particle size: 1.8 μm, length: 100 mm, and internal diameter: 2.1 mm Palo Alto, CA, United States). The column oven, mobile gradient, and electrospray ionization technique used were as described previously (Jiang et al., 2013). (-)-EC, (-)-ECG, (++)-C, (-)-EGC, (-)-EGCG, rutin, and chlorogenic acid standards that were purchased from Sigma (St. Louis, MO, United States) were used positive controls.

HPLC analysis for amino acids was carried out on a Waters 600 HPLC equipped with a binary 2489 UV detector (Milford, MA, United States). A C18 reverse phase column (Nova-Pak, 250 mm, 34.6 mm, particle size 51 mm) was used to separate amino acids at 37°C. The excitation and emission wavelengths of the detector were 250 nm and 395 nm, respectively. The UV-visible detector wavelength was set at 248 nm, and the UV detector wavelength was set at 248 nm. An elution gradient program was used to separate metabolites according to a previously published method (Zhang et al., 2010).

For UPLC-MS/MS and HPLC analyses, three technical replicates were completed for each sample. The mean values of three replicates were calculated and statistically evaluated by Student *t*-test using the SPSS software.

### Statistic Methods

All assays described above were performed with either three repetitive experiments or three biological replicates. Mean values were calculated and then statistically evaluated by Student *t*-test using the SPSS software. The Pearson Correlation Coefficient (PCC) analysis using the SPSS software was also performed to evaluate correlation between *CsMYB4a* expression level, the total phenolic acid contents, total tea catechin contents, and contents of two main individual compounds. Test of significance was the two-tailed type. *P*-values less than 0.05 were evaluated to be statistically significant.

## Results

### Cloning of *CsMYB4a* and Sub-cellular Localization

**FIGURE 1:**
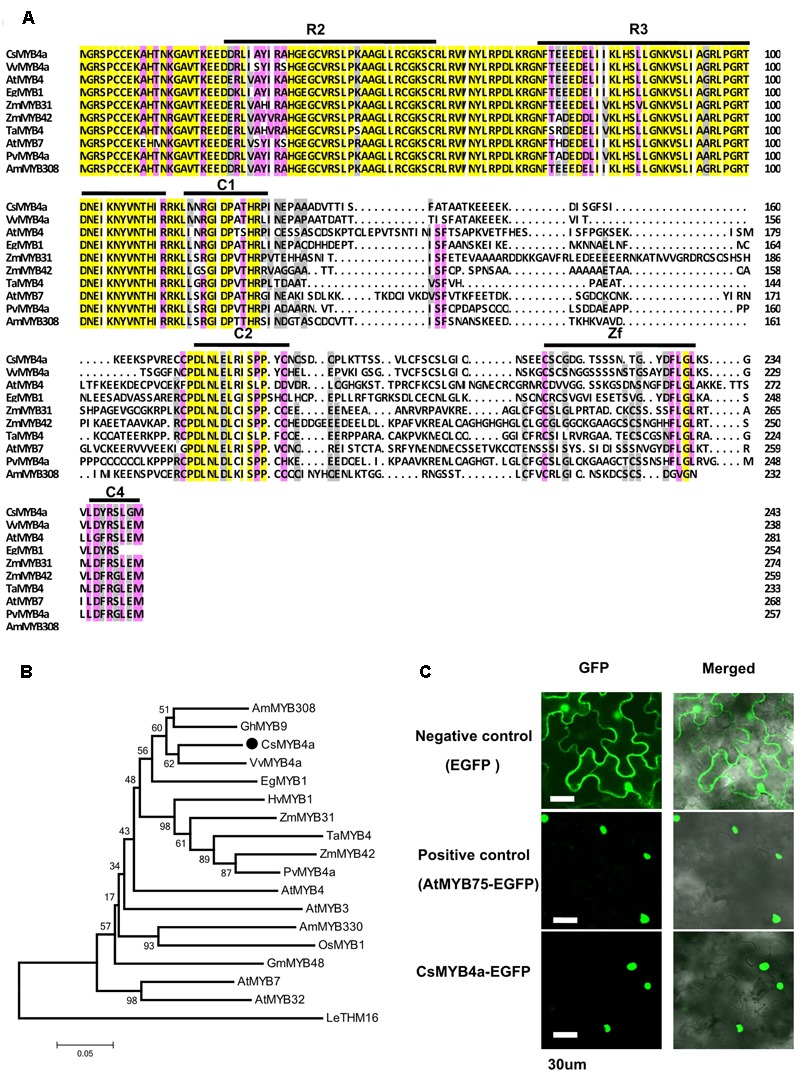
Amino acid sequence alignment and phylogenetic analysis of CsMYB4a. **(A)** Amino acid sequence alignment utilized CsMYB4a and 10 other R2R3 MYB4 subgroup members. Black boxes and red boxes highlight the R2R3 domains and the potential regulatory domains, respectively. **(B)** A phylogenetic tree resulting from MEGA analysis using amino acid sequences of CsMYB4a and 18 other R2R3 MYB4 subgroup members. Accession numbers for MYB4-sugroup sequences: VvMYB4a (NP_001268129.1), EgMYB1 (CAE09058), AmMYB308 (JQ0960), ZmMYB31 (CAJ42202), ZmMYB42 (CAJ42204), TaMYB4 (AAT37167), PvMYB4a (JF299185), AtMYB4 (AY519615), AtMYB7 (AEC06531), AtMYB32 (NP_195225), AtMYB3 (NP_564176), AmMYB330 (P81395), GhMYB9 (AAK19619), GmMYB48 (ABH02823), OsMYB1 (BAA23337) and HvMYB1 (P20026). **(C)** Images obtained using confocal microscopy indicated nuclear localization of the fused CsMYB4a-eGFP protein in leaf epidermal cells of *Nicotiana benthamiana*. AtMYB75–eGFP and eGFP alone were used as controls. Bar = 30 μm.

In our previous transcriptomic study, 73 tea cDNAs were annotated to encode R2R3-MYB transcription factors. One of the cDNA sequences, namely *CsMYB4a* (previously named as *CsMYB4-6*), was identified as a member of the MYB4 subgroup (Zhao et al., 2013). Based on its nucleotide sequence, a full length ORF consisting of 735 bp was amplified from young leaves and was deduced to encode 244 amino acids (**Figure 1A**). An alignment of amino acid sequences of CsMYB4a and eight other homologs revealed that it shared 60.1, 56, and 55% identity with VvMYB4a, AtMYB4, and EgMYB1, respectively (**Figure 1A**). This alignment further determined that CsMYB4a contained the R2 and R3 conserved motifs in the N-terminal region, and the C1, C2, Zf, and C4 conserved motifs in the C-terminal region (**Figure 1A**). CsMYB4a also possesses the conserved motif DLNLEL, which is located within the C2 motif and is a sequence feature of the MYB4 subgroup. A phylogenetic analysis was performed using the amino acid sequences of CsMYB4a and 14 other homologs. The resulting unrooted tree indicated that CsMYB4a was clustered together in the same clade as VvMYB4a, EgMYB1, and AmMYB308 (**Figure 1B**), which are members of theMYB4 subgroup and repressors of the phenylpropanoid pathway. These data suggested that CsMYB4a might negatively regulate the phenylpropanoid pathway in tea plants.

GFP fusion is an efficient approach for subcellular localization of transcription factors (Unver et al., 2013). In order to identify the subcellular localization of CsMYB4a, an eGFP ORF was fused to the C-terminus of its ORF fragment and then introduced into *N. benthamiana*. In addition, positive (fused AtMYB75-eGFP) and negative controls (eGFP) were introduced into *N. benthamiana*. Confocal microscopy observation determined that the CsMYB4a-eGFP fusion protein was localized in the nucleus (**Figure 1C**).

### Relevance between the Expression Profile of *CsMYB4a* and the Content of Phenylpropanoid Compounds in Tea Tissues

**FIGURE 2:**
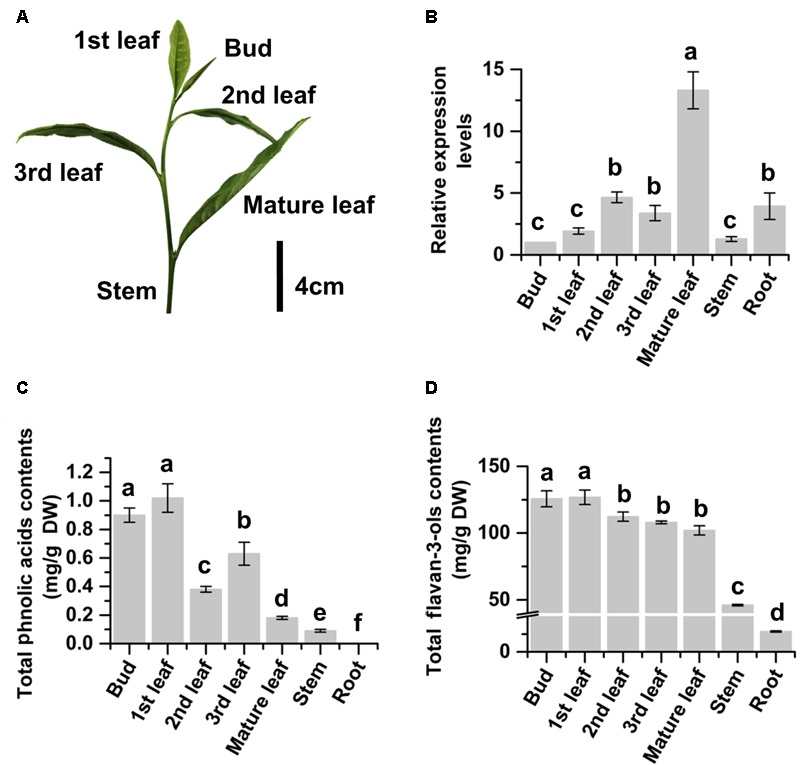
Relative expression levels of *CsMYB4a* and contents of phenylpropanoid metabolites in different organs of tea plants. **(A)** A picture shows a bud and different developmental stages of leaves (1st, 2nd, 3rd, and mature) collected from multiple plants for gene expression and phenylpropanoid metabolite analyses. **(B)** qRT-PCR data show expression profiles of *CsMYB4a* in buds, leaves (different stages), young stems, and roots. **(C)** Total phenolic acid’s contents summed from the contents of both caffeoylquinic acid and p-coumaroylquinic acid in buds, leaves (different stages), young stems, and roots. **(D)** Total flavan-3-ols contents in buds, leaves (different stages), young stems, and roots. Values represent the mean ± SD (*n* = 3). The lowercase “a, b, c, d” labeled on top of bars indicate that those values are significantly different from that of the equivalent control in **B–D**.

The expression profile of *CsMYB4a* in tea was characterized with qRT-PCR analysis. The resulting data showed that the expression level of *CsMYB4a* followed an increase trend from the top buds through the 1st and 2nd leaves to the fully expanded mature leaves at the lower positions (**Figures 2A,B**). The expression level in mature leaves was approximately 12.5 times higher than that in buds (**Figure 2B**). To understand whether its expression profile was associated with the accumulation of primary tea phenylpropanoids, UPLC-MS analysis was carried out to quantify flavan-3-ols and phenolic acids. (-)-Epicatechin, (-)-epigallocatechin, (-)-epigallocatechin-3-gallate, (-)-epicatechin-3-gallate, and catechin are tea flavan-3-ols. Quantification of total contents of these compounds showed that the contents of these compounds were similar in the top buds and the 1st leaf but significantly decreased in the lower mature leaves (**Figure 2C**). The similar decrease trend was observed for total contents of two phenolic acids, caffeoylquinic acid and p-coumaroylquinic acid. These data showed a negatively correlation between the gene expression of *CsMYB4a* and these tea phenylpropanoid compounds. In addition, PCC analysis revealed that the expression levels of *CsMYB4a* were significantly negatively associated with the contents of total phenolic acids, p-coumaroylquinic acid, and epicatechin-gallate (Supplementary Table S2). The negative correlation between the expression levels of *CsMYB4a* and total catechins was nearly significant (*P*-value, 0.08).

### CsMYB4a Binds Promoters of Five Phenylpropanoid Pathway Genes in Tea Plants

**FIGURE 3:**
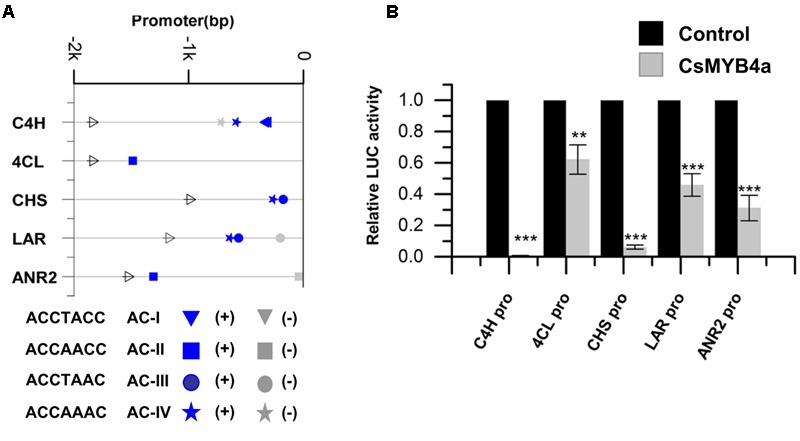
CsMYB4a repressed the promoter activity of genes involved in the phenylpropanoid and flavonoid biosynthesis pathways. **(A)** Distribution of the four classes of AC-elements in the promoters of genes involved in phenylpropanoid and flavonoid biosynthesis pathways in tea plants. Sense elements and anti-sense elements are represented in blue and gray, respectively. **(B)** Dual luciferase (LUC) assays of CsMYB4a (effector) and the promoters fused to firefly luciferase (reporter). Empty vector was used as the effector in the control assay. The promoters of *C4H, 4CL, CHS, LAR*, and *ANR2* genes were used in dual luciferase assays (*n* = 3, ^∗^*P* < 0.05, ^∗∗^*P* < 0.01, ^∗∗∗^*P* < 0.001).

The promoter sequences of five genes, *CsC4H, Cs4CL, CsCHS, CsLAR*, and *CsANR2*, were isolated from tea plants using a reported genome-walker protocol (Vineet Kumar et al., 2014). The length of 1–2 kb nucleotides in the upstream of the coding region of these genes were cloned from leaves. Sequence analysis identified four types of AC-elements, AC-I: ACCTACC, AC-II: ACCAACC, AC-III: ACCTAAC, and AC-IV: ACCAAAC (**Figure 3A**). The promoter sequences of *CsC4H, Cs4CL, CsCHS, CsLAR*, and *CsANR2* contain AC-I and AC-IV, AC-II alone, AC-III and AC-IV, AC-III and AC-IV, and AC-II alone, respectively (**Figure 3A**).

To demonstrate whether or not CsMYB4a represses the promoter activity of *CsC4H, Cs4CL, CsCHS, CsLAR*, and *CsANR2* genes, the interaction of CsMYB4a with these five genes’ promoters was analyzed *in vitro*. Each promoter was cloned into the PGWL7 vector to obtain new vectors, PGWL7-pCsC4H, -pCs4CL, -pCsCHS, -pCsLAR, and -pCsANR2, in each plasmid of which each gene’s promoter was placed to drive a luciferase reporter gene. The resulting promoter-luc cassette was used as an effector of CsMYB4a. The co-expression of *CsMYB4a* and each effector vector was analyzed in *Arabidopsis* protoplasts using a dual-luciferase assay. The resulting data showed that CsMYB4a significantly reduced the level of the luciferase signal controlled by each promoter (**Figure 3B**). In particular, the luciferase signals controlled by the promoter of *CsC4H* and *CsCHS* were reduced by approximately 99 and 94%, respectively (**Figure 3B**). These results clearly indicated that CsMYB4a repressed the promoting activity of the *CsC4H, Cs4CL, CsCHS, CsLAR*, and *CsANR2* promoters.

### Overexpression of *CsMYB4a* Represses Tobacco Growth and Development

**FIGURE 4:**
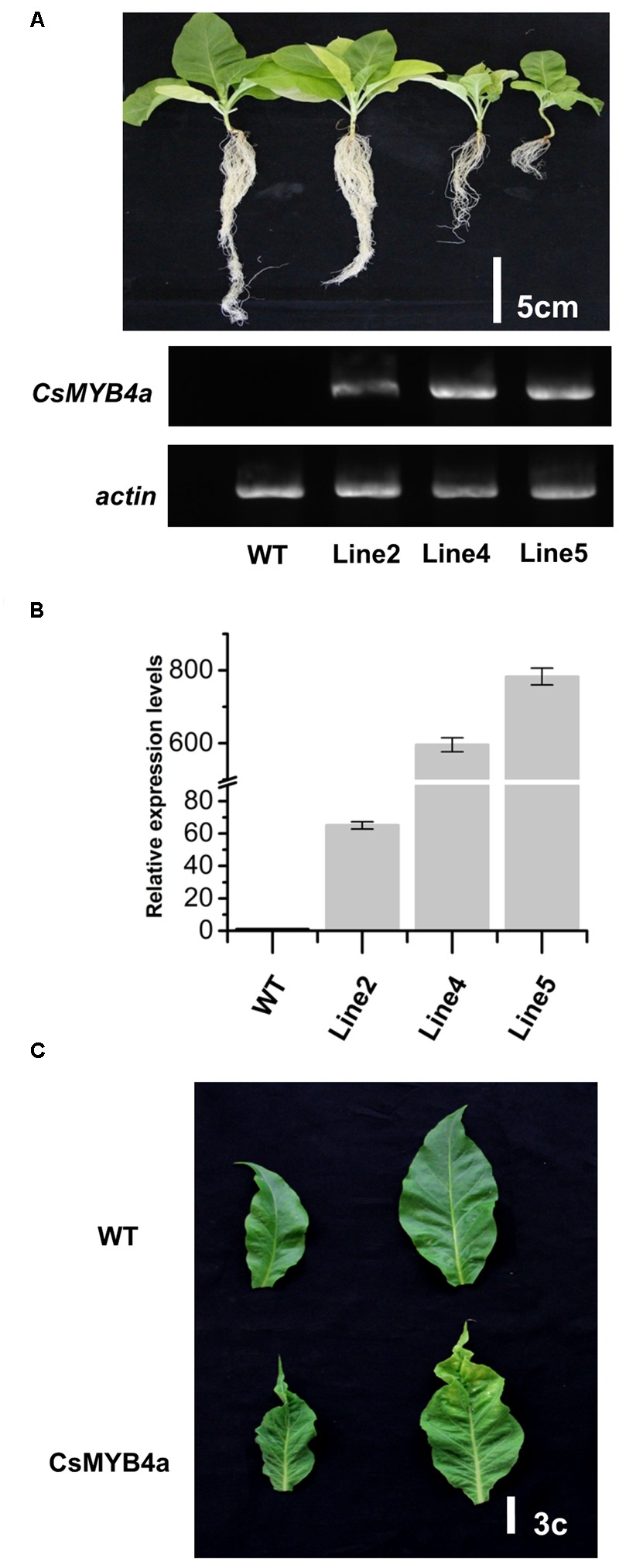
Phenotypic comparison of *CsMYB4a* transgenic and wild-type *N. tabacum* plants. **(A)** Phenotypes of 50-day old T1 progenies and semi-quantitative PCR analysis of 3 different T1 generation *CsMYB4a* transgenic tobacco lines (line2, line4, and line5) and wild-type tobacco plants. **(B)** qRT-PCR analysis of the three different *CsMYB4a* transgenic tobacco lines (2, 4, and 5). Values represent the mean ± SD (*n* = 3). **(C)** The leaf lesion phenotypes of T1 generation *CsMYB4a* transgenic tobacco (line2, line4 and line5) and wild-type plants.

The full length ORF of *CsMYB4a* was overexpressed in tobacco plants and multiple candidate transgenic plants were obtained using antibiotic selection (Supplementary Figure S3A). Seeds of T0 plants were harvested and grown on soil to obtain T1 progeny (**Figure 4A**). Transgenic progeny exhibited abnormal growth as compared to wild-type plants grown in the same glass house. Transgenic plants were characterized by dwarfing, small leaves, leaf shrinkage, chlorosis, pale coloration, yellowish patches on leaves, and early senescence (**Figures 4A,C** and Supplementary Figure S3). It was clearly indicated that the heights of transgenic plants were significantly reduced in comparison with wild-type plants. In addition, the development of lateral roots was severely inhibited in transgenic lines (**Figure 4A**). Both semi-quantitative and qRT-PCR analyses demonstrated that the *CsMYB4a* transgene was highly expressed in selected lines of T1 plants (**Figures 4A,B**), confirming the correlation of the transgene expression with the observed alterations in plant morphology.

### Genome-Wide Transcriptomic Analysis of Transgenic vs. Wild-Type Plants

Genome-wide transcriptomics is routinely utilized to understand targeted genes transcriptionally associated with plant metabolism (Gurkok et al., 2015). In our study, genome-wide transcriptomic analysis was conducted using 30-day-old seedlings of T1 progeny of *CsMYB4a* transgenic (line 4) and wild-type tobacco plants (Supplementary Figure S3B). Transgenic progeny were characterized by dwarfing, yellow leaves, short and less lateral roots. Sequence assembly obtained 52,290 unigenes, with an average length of 778 bp nucleotides (Supplementary Table S3) and high quality sequence data. Gene annotation was conducted using NR, NT, Swiss-Prot, KEGG, COG, and GO databases. As a result, 39,313 unigenes were annotated (Supplementary Table S4). In addition, values for Fragments Per kb per Million Reads (FPKM) were calculated to understand the expression levels of all unigenes. The resulting FPKM values allowed identifying unigenes that were differentially expressed in the transgenic vs. WT plants. Based on a FPKM ratio value greater or less than 2-fold (*P*-value ≤ 10^-5^ and FDR ≤ 0.001), 5,765 genes were identified to be differentially expressed in *CsMYB4a* transgenic plants, of which 2,584 were up-regulated and 3,181 were down-regulated (Supplementary Figure S4). GO annotations revealed 49 different classes of functions (Supplementary Figure S5). Further KEGG pathway enrichment analysis (Kanehisa et al., 2008) characterized that 5,765 differently expressed genes were involved in 25 different categories of plant metabolisms, two main pathways of which are associated with the biosynthesis of phenylpropanoids and phenylalanine (Supplementary Table S5).

### Overexpression of *CsMYB4a* Suppresses the Expression of the Phenylpropanoid Pathway Genes and Leads to Reduction of Lignin and Other Compounds

**FIGURE 5:**
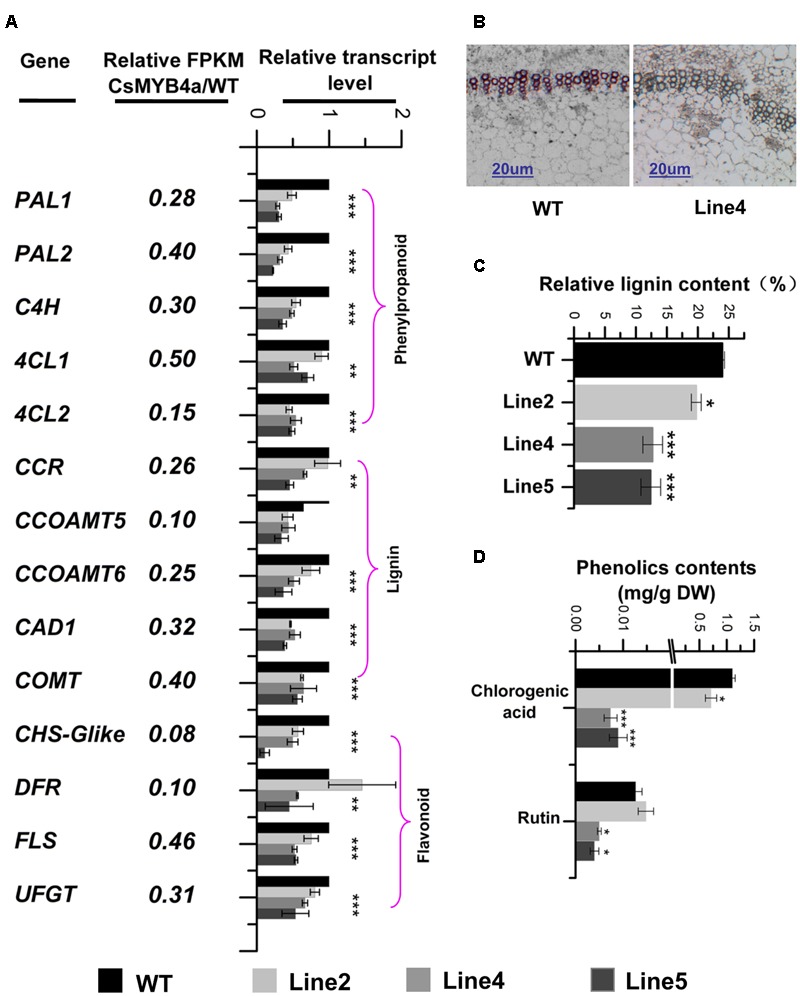
Effect of *CsMYB4a* overexpression on the transcriptional level of 14 phenylpropanoid pathway genes and the level of lignin, chlorogenic acid and rutin. **(A)** Ratio values were obtained by dividing each gene RPKM value in *CsMYB4a* transgenic plants by its RPKM value in wild-type plants. qRT-PCR analysis of each gene in three different transgenic lines and wild-type plants. **(B)** Cross-sectional images of stems in wild-type and *CsMYB4a* transgenic tobacco plants revealing lignin via phloroglucinol-HCl staining. **(C)** Relative lignin content (proportional to dry weight) of wild-type plants and different lines of *CsMYB4a* transgenic tobacco plants. **(D)** Level of chlorogenic acid and rutin in seedlings of wild-type and *CsMYB4a* transgenic plants. Values represent the mean ± SD (*n* = 3). Asterisks on top of the bars indicate that the values are significantly different from the equivalent control (^∗^*P* < 0.05, ^∗∗^*P* < 0.01, ^∗∗∗^*P* < 0.001).

Further data mining determined that the FPKM values for *PAL* (two members), *C4H, 4CL (two members), CCR, CCOAMT* (two members), *COMT, CAD, CHS, DFR, FLS*, and *UFGT* were significantly lower in transgenic plants than in wild-type plants (**Figure 5A**). These transcriptional alterations were confirmed by qRT-PCR analysis of three transgenic lines (lines 2, 4, 5) (**Figure 5A**). The resulting data indicated that transcript levels of *PAL1, PAL2, C4H, 4CL1, 4CL2, CCR, CCOAMT5, CCOAMT6, CAD, COMT, CHS, DFR, FLS* and *UFGT* were reduced by 50 to 92% in *CsMYB4a* transgenic plants (**Figure 5A**).

Lignin that represents one main group of polymeric end-products of the phenylpropanoid pathway is essentially associated with mechanical support, cell wall structure, and plant growth. Phloroglucinol staining analysis revealed that the lignin staining in the xylem of transgenic plants was much lighter than in those of wild-type plants (**Figure 5B**). An estimation of total lignin was also obtained using a spectrophotometer-based analysis. Relative to wild-type plants, the total lignin content in *CsMYB4a* transgenic plants was significantly reduced by 5 to 13% (**Figure 5C**). Two other phenylpropanoid compounds, chlorogenic acid and rutin, were also measured using UPLC-MS. In *CsMYB4a* transgenic lines 4 and 5, the content of chlorogenic acid was reduced by 99 and 98% and the rutin contents was reduced by approximately 55 and 65% (**Figure 5D**).

### Overexpression of *CsMYB4a* Down-regulates the Shikimate Pathway

**FIGURE 6:**
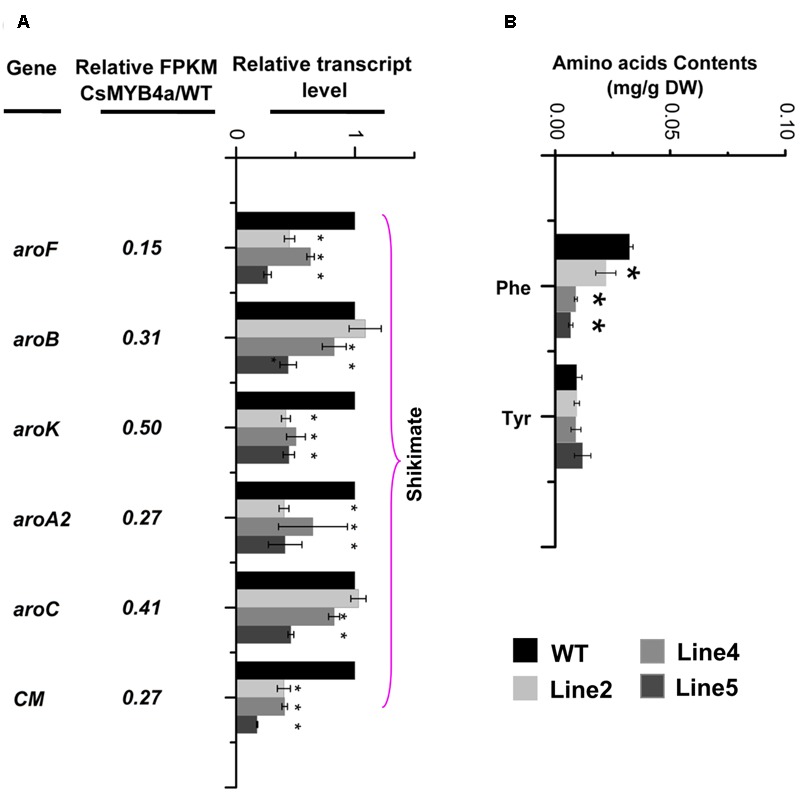
*CsMYB4a* overexpression affects the transcriptional level of six shikimate pathway genes and amino acid levels. **(A)** Ratio values were obtained by dividing the RPKM value of each gene in *CsMYB4a* transgenic plants by the corresponding RPKM value in wild-type plants. qRT-PCR analysis of each gene in three different lines of transgenic plants and wild-type plants. **(B)** Level of different amino acids in wild-type and *CsMYB4a* transgenic tobacco plants. Values represent the mean ± SD (*n* = 3). Asterisks on top of the bars indicate significant differences from the equivalent control (^∗^*P* < 0.05, ^∗∗^*P* < 0.01, ^∗∗∗^*P* < 0.001).

L-phenylalanine is synthesized via the shikimate pathway. Sequence analysis identified that the FPKM values of *aroF, aroB, aroK, aroA2, aroC*, and *CM* were significantly lower in transgenic plants relative to their values in wild-type plants (**Figure 6A**). qRT-PCR analysis confirmed that the expression level of these genes was significantly reduced in transgenic plants (**Figure 6A**). HPLC analysis determined that the content of L-phenylalanine was reduced by 30–60% in three of the *CsMYB4a* transgenic lines (**Figure 6B**).

### Characterization of AC-Elements in Promoters of 20 Phenylpropanoid and Shikimate Pathway’s Genes and Analysis of CsMYB4a Binding

**FIGURE 7:**
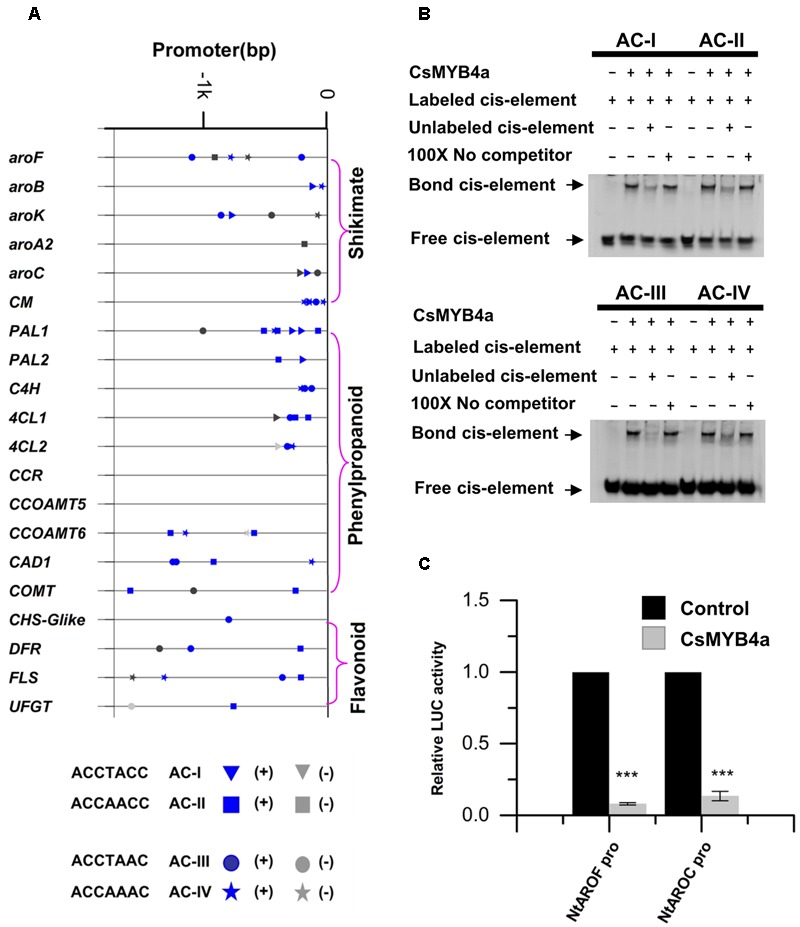
Analysis of CsMYB4a binding AC-elements of promoters. **(A)** A diagram indicating the location of four AC-elements in the promoter region of selected differentially expressed tobacco genes. **(B)** EMSA assay of CsMYB4a protein possessing four AC-elements. Salmon sperm DNA was used as a non-specific competitor. **(C)** Dual luciferase (LUC) assays of CsMYB4a (effector) and the promoters fused to firefly luciferase (reporter). Empty vector was used as the effector in the control assay. The promoters of *NtAROF, NtAROC*, were used in the dual luciferase assay (*n* = 3, ^∗^*P* < 0.05, ^∗∗^*P* < 0.01, ^∗∗∗^*P* < 0.001).

MYB4 subgroup members, such as AtMYB4, EgMYB1, and ZmMYB31, have been demonstrated to bind AC-elements of promoters to repress certain types of plant natural product biosynthesis such as phenylpropanoid biosynthesis (Legay et al., 2007; Zhao et al., 2007; Fornalé et al., 2010). To determine whether or not the down-regulation of gene expression described above was associated with CsMYB4a binding, promoter nucleotide sequences of 20 tobacco genes were identified from tobacco genomic sequences curated at NCBI. All promoter sequences were 1.6 kb in length. Analysis of these promoter sequences identified four types of AC elements, type I: ACCTACC, type II: ACCAACC, type III: ACCTAAC, and type IV: ACCAAAC (**Figure 7**). The promoter sequences of six shikimate pathway genes, *aroF, aroB, aroK, aroA2, aroC*, and *CM*, also contained at least one type of AC-elements. The promoter sequences of 13 phenylpropanoid pathway genes, including five early pathway genes (*PAL1, PAL2, C4H, 4CL1*, and *4CL2*), three lignin pathway genes (*CCOMT6, CAD1*, and *COMT*), and three flavonoid pathway genes (*CHS-Glike, DFR*, and *FLS*), also possessed at least one type of AC-elements (**Figure 7A**). The presence of the AC-elements suggested that CsMYB4a could theoretically bind these promoters.

Electrophoretic mobility shift assay experiments were carried out to verify whether or not CsMYB4a could bind those AC-element-containing promoters. *CsMYB4a* was cloned into the PRSF prokaryotic expression vector to obtain a new plasmid, namely PRSF-CSMYB4a. In this plasmid, a His-tag was fused to the N-terminal region of CsMYB4a. The EMSA results demonstrated that the recombinant CsMYB4a protein could directly *in vitro* bind probes containing the sequences of AC-I, AC-II, AC-III, and AC-IV (**Figure 7B**). In addition, competitor and non-competitor probe controls were used in the EMSA experiments. Results demonstrated that the binding capacity of the recombinant CsMYB4a was much less efficient in the presence of a competitor probe. These data demonstrated the binding capacity of CsMYB4a to AC-elements in 20 promoters.

A dual-luciferase assay was particularly conducted to verify the binding capacity of CsMYB4a to promoters containing AC-elements. Candidates tested in experiments included the promoters of *NtAROF* and *NtAROC* controlling the shikimate pathway. Based on the intensity of the luciferase signal, the promoting activity of *NtAROF* and *NtAROC* promoters were reduced by 96 and 87% (**Figure 7C**), respectively. These results showed that CsMYB4a bound to the promoters containing AC-elements, thus repressed their promoting activity.

## Discussion

### CsMYB4a Is a Repressive Transcription Factor of the Phenylpropanoid Pathway

Members of the MYB4 subgroup have been characterized to repress the phenylpropanoid pathway. The MYB4 subgroup is featured by R2R3, C1, C2, Zf, and C4 domains, which have been identified in both monocot and dicot plants (including herbaceous and woody plants) (**Figure 1**; Kranz et al., 1998; Tamagnone et al., 1998; Jin et al., 2000; Nakachi et al., 2000; Fornalé et al., 2006, 2010; Legay et al., 2007; Sonbol et al., 2009; Dubos et al., 2010; Ma et al., 2011). The C1 domain has been reported to potentially activate gene expression, while the C2 and C4 domains have been reported to repress gene expression. These observations suggest that the C1, C2, and C4 domains are likely associated with the different specificity and regulation activity exhibited by MYB4 members reported in various plant species (Tamagnone et al., 1998; Shen et al., 2012; Zhou et al., 2015). Another regulation feature of the MYB4 members is the binding activity to four types of AC-elements. In present report, we isolated *CsMYB4a* encoding a R2R3MYB transcription factor that belongs to the MYB4 subgroup. The expression profile of *CsMYB4a* showed negative correlation to the accumulations of tea flavan-3-ols and two phenolic acids (**Figure 2** and Supplementary Table S2). We hypothesized that *CsMYB4a* might encode a repressor that down-regulated the formation of phenylpropanoid compounds in tea plants. Although functional analysis for tea genes of interests is very challenging in this species due to recalcitrant regeneration and no success in genetic transformation and lacking appropriate genetic materials, such as mutants, we used an integrative approach to understand the CsMYB4a’s regulation activity. Sequence analysis revealed that CsMYB4a contains R2R3, C1, C2, Zf, and C4 domains. In order to demonstrate whether or not CsMYB42 could bind to AC-elements, the promoter sequences of 5 tea genes (*CsC4H, Cs4CL, CsCHS, CsLAR*, and *CsANR2*) were isolated from tea and used for an *in vitro* analysis. Sequence analysis determined that five promoters contain one or two AC-elements (**Figure 3A**). Furthermore, CsMYB4a and promoter binding analysis using a dual-luciferase as a reporter verified that CsMYB4a significantly decreased the promoting activity of these five promoters (**Figure 3B**). These data support the premise that CsMYB4a functions as a repressive transcription factor of the phenylpropanoid pathway in tea plants.

To further determine whether or not *CsMYB4a* could repressively control the phenylpropanoid pathway, its ORF was overexpressed in tobacco plants. Transgenic analysis demonstrated that the overexpression of *CsMYB4a* altered growth of transgenic tobacco (**Figure 4**). A genome-wide transcriptomic study disclosed that the FPKM values of 14 phenylpropanoid pathway genes controlling the formation of lignin and flavonoid biosynthesis were significantly lower in transgenic plants as compared to WT (**Figure 5A**). The results of qRT-PCR analysis further supported that the expression levels of these genes were significantly reduced in transgenic plants compared to wild plants (**Figure 5A**). Correspondingly, the content of lignin, chlorogenic acid, and rutin were significantly lower in those transgenic plants than in WT. The repressive functionality of CsMYB4a was further supported by an analysis of promoter sequences. Four types of AC-elements were characterized in the promoters of 14 phenylpropanoid pathway genes in tobacco plants. These AC-elements serve as binding targets for all of the reported MYB4 members that repress phenylpropanoid biosynthesis (Romero et al., 1998; Legay et al., 2007; Zhao et al., 2007; Shen et al., 2012). For example, AmMYB330, AmMYB308, AtMYB4, ZmMYB31, ZmMYB42, TaMYB4, EgMYB1, and PvMYB4 have been demonstrated to bind at least one of the AC-elements to negatively regulate lignin formation or the biosynthesis of other phenylpropanoid compounds (Tamagnone et al., 1998; Jin et al., 2000; Fornalé et al., 2006, 2010; Legay et al., 2007; Sonbol et al., 2009; Ma et al., 2011; Shen et al., 2012). In the present study, 14 promoter sequences of phenylpropanoid pathway genes, *PAL1, PAL2, C4H, 4CL1, 4CL2, CAD1, CCR, CCoAMT1, CCoAMT2, COMT, CHS-G-like, DFR, FLS*, and *UFGT*, were identified from tobacco plants. All of these promoters possess at least one type of AC-element (**Figure 7A**). Furthermore, EMSA experiments demonstrated that CsMYB4a could bind all four types of AC-elements (**Figure 7B**). These data indicated that the ectopically expressed CsMYB4a bound to the promoters of these genes and repressed their promoting activity. These data support the premise that CsMYB4a represses the phenylpropanoid pathway.

### CsMYB4a Repressively Controls the Shikimate Pathway

Few studies have shown that the MYB4 subgroup negatively regulates gene expression involved in the shikimate pathway. The *aroF* gene encodes 3-deoxy-d-arabino-heptulosonate 7-phosphate synthase (DHS2), which catalyzes the first step of the shikimate pathway (Supplementary Figure S6). In white spruce [*P. (Moench) Voss*], its promoter sequence was recently characterized to contain AC-I and AC-II elements, which are targets of two R2R3-MYB members. Two R2R3-MYB transcription factors, namely PgMYB14 and PgMYB15, were demonstrated to regulate the expression of PgaroF (encoding *PgDHS2*) via binding AC-elements (Bomal et al., 2015). Another recent study discovered that an R2R3-MYB transcription factor from *Populus*, namely MYB182, repressively regulated the shikimate, anthocyanin, and proanthocyanidin pathways (Yoshida et al., 2015). When *MYB182* was overexpressed in transgenic *Populus* plants, the expression of *aroF, aroA, aroC*, and *CM* was significantly reduced. In our experiments, when *CsMYB4a* was overexpressed in tobacco plants, genome-wide transcriptomic and qRT-PCR analyses revealed that the expression level of six shikimate pathway genes controlling the biosynthesis of Phe (Supplementary Figure S6) were significantly downregulated in transgenic plants (**Figure 6A**). Further sequence analysis revealed the presence of AC-elements in the promoters of these genes, indicating that CsMYB4a could bind these promoters, thus inhibited their activity. A dual-luciferase assay further verified that the activity of *AROF* and *AROC* promoters was strongly reduced by CsMYB4a (**Figure 7C**). Further metabolic analyses confirmed that the level of phenylalanine (Phe) was significantly reduced in three different transgenic lines. All these data showed that the *CsMYB4a* transgene repressively regulated the shikimate pathway in tobacco plants. In addition, it was interesting that our metabolic pathway enrichment analysis revealed differential gene expression patterns associated with plant hormone signal transduction and diterpenoid metabolism in *CsMYB4a* transgenic tobacco plants. These transcriptional data infers potential unknown regulatory functions of CsMYB4a in plants for our future investigations.

## Conclusion

**FIGURE 8:**
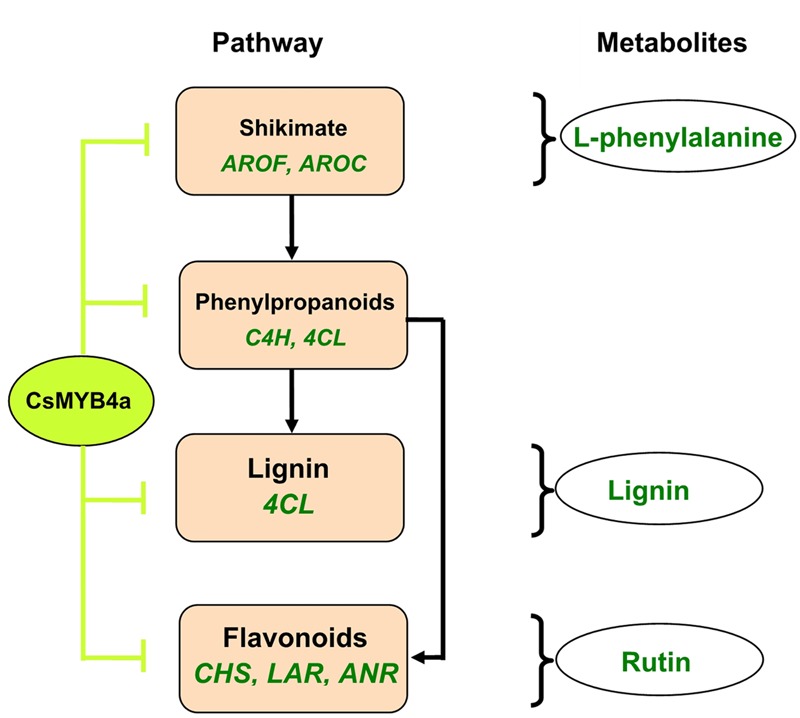
A simplified scheme showing repressive effects of the CsMYB4a on the shikimate and phenylpropanoid pathways. CsMYB4a represses promoting activities via targeting promoters of two genes (*AROF* and *AROC*) in the shikimate pathway and of five genes (*C4H, 4CL, CHS, LAR*, and *ANR*) in the phenylpropanoid pathway. Its repressive activity leads to reduction of phenylalanine, lignin, and flavonoids.

This study using an integrative approach demonstrates that CsMYB4a negatively regulates the biosynthesis of phenylalanine, phenolic acids, lignin, and flavonoids. It inhibits the promoter activity of two shikimate pathway genes (*AROF* and *AROC*) and of five phenylpropanoid pathway genes (*C4H, 4CL, CHS, LAR*, and *ANR*) (**Figure 8**). It is a repressive R2R3-MYB transcription factor of the shikimate, lignin, and flavonoid pathways.

## Author Contributions

ML designed the study, performed the experiments, interpreted the results, and wrote the manuscript. LGu and NG contributed to the plant genetic transformation. YzL and YW participated in the cloning of the promoters. WJ and YP participated in the dual-luciferase essays. LZ participated in the Race cloning. XJ and YjL contributed to the HPLC and UPLC-MS analysis. LGa, D-YX, and TX designed the experiments, discussed the results, edited the manuscript and supervised the project.

## Conflict of Interest Statement

The authors declare that the research was conducted in the absence of any commercial or financial relationships that could be construed as a potential conflict of interest.

## Footnotes

1 http://www.genomics.cn/index

2 http://www.ncbi.nlm.nih.gov/

3 http://plantpan2.itps.ncku.edu.tw/
