# Supplementary material for: Functional Characterization of Tea (Camellia sinensis) MYB4a Transcription Factor Using an Integrative Approach
Source: Front Plant Sci. 2017 Jun 12;8:943. doi: 10.3389/fpls.2017.00943 (PMC5467005; doi:10.3389/fpls.2017.00943)
Supplement: Supplementary file 1 [file Data_Sheet_1.PDF]

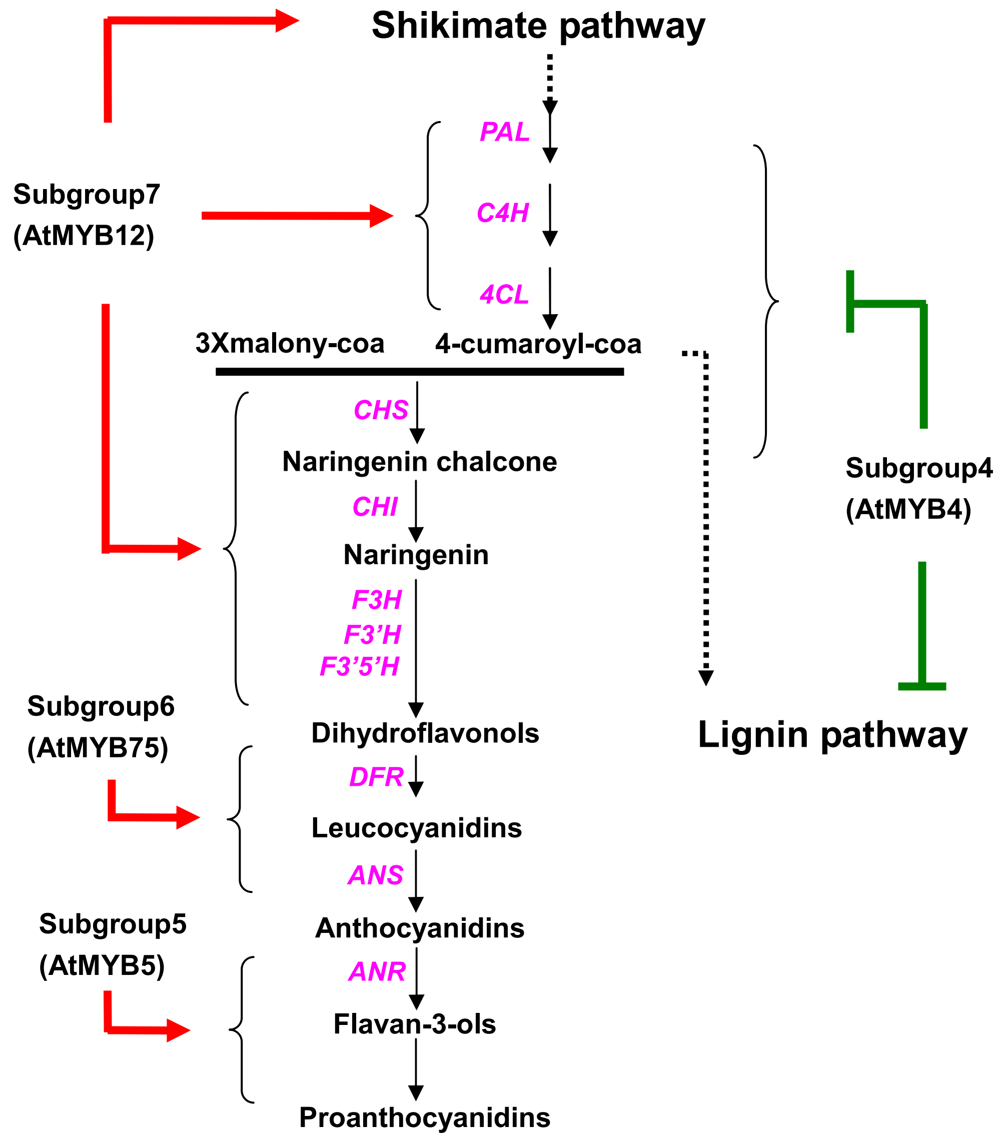

Figure S1. Regulation of the shikimate and phenylpropanoid pathways by different R2R3-MYB subgroups in plants.

The green solid lines indicate that the MYB4 subgroup members represses these pathways, while the red solid lines indicate that the MYB5, 6 and 7 subgroups members activate these pathways. Structural genes are highlighted with pinkish color.

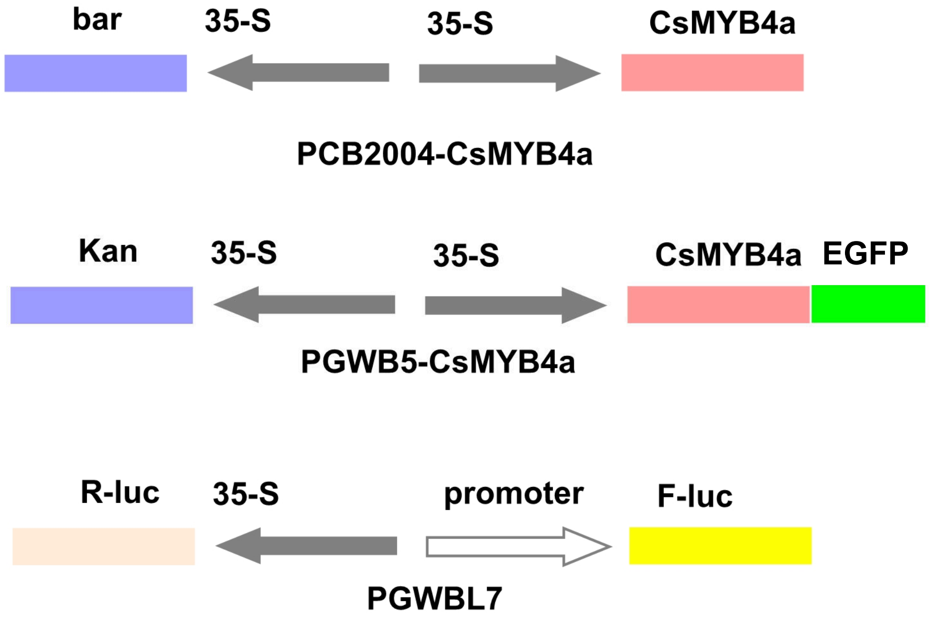

Figure S2. Maps of vectors developed for genetic transformation of tobacco plants for overexpression (top), subcellular localization of protein (middle), and dual-luciferase assay (bottom).

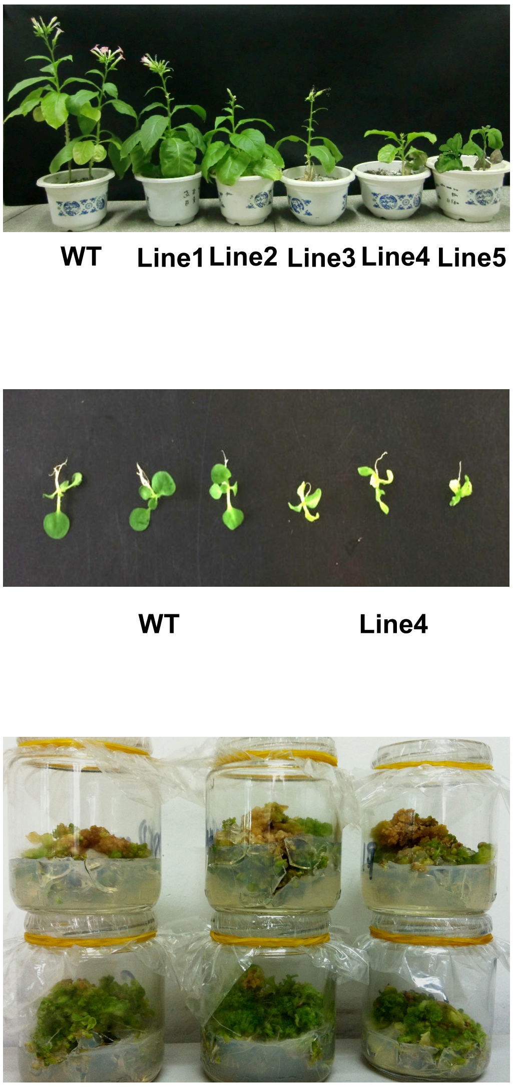

Figure S3. Phenotypes of CsMYB4a transgenic versus wild-type (WT) tobacco plants. (A) Growth phenotypes of 90-day old T0 *CsMYB4a* transgenic tobacco lines and WT tobacco plants; (B) Phenotypes of 30-day-old T1 progeny seedlings of *CsMYB4a* transgenic line4 and WT tobacco plants, which were used for transcriptomic analysis; (C) Different phenotypes of callus induction from T1 progenies of transgenic line 4 and WT plants.

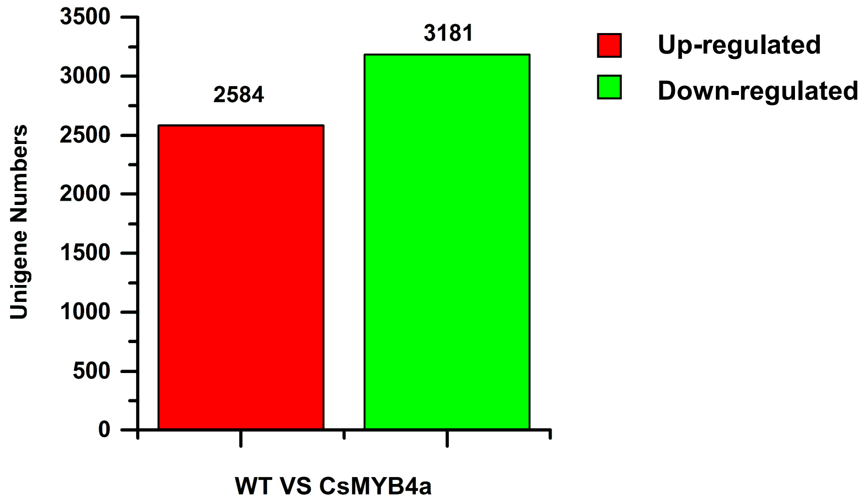

Figure S4. Numbers of differentially expressed genes identified from sequencing. The red and green columns show the number of up-regulated and down-regulated genes, respectively.

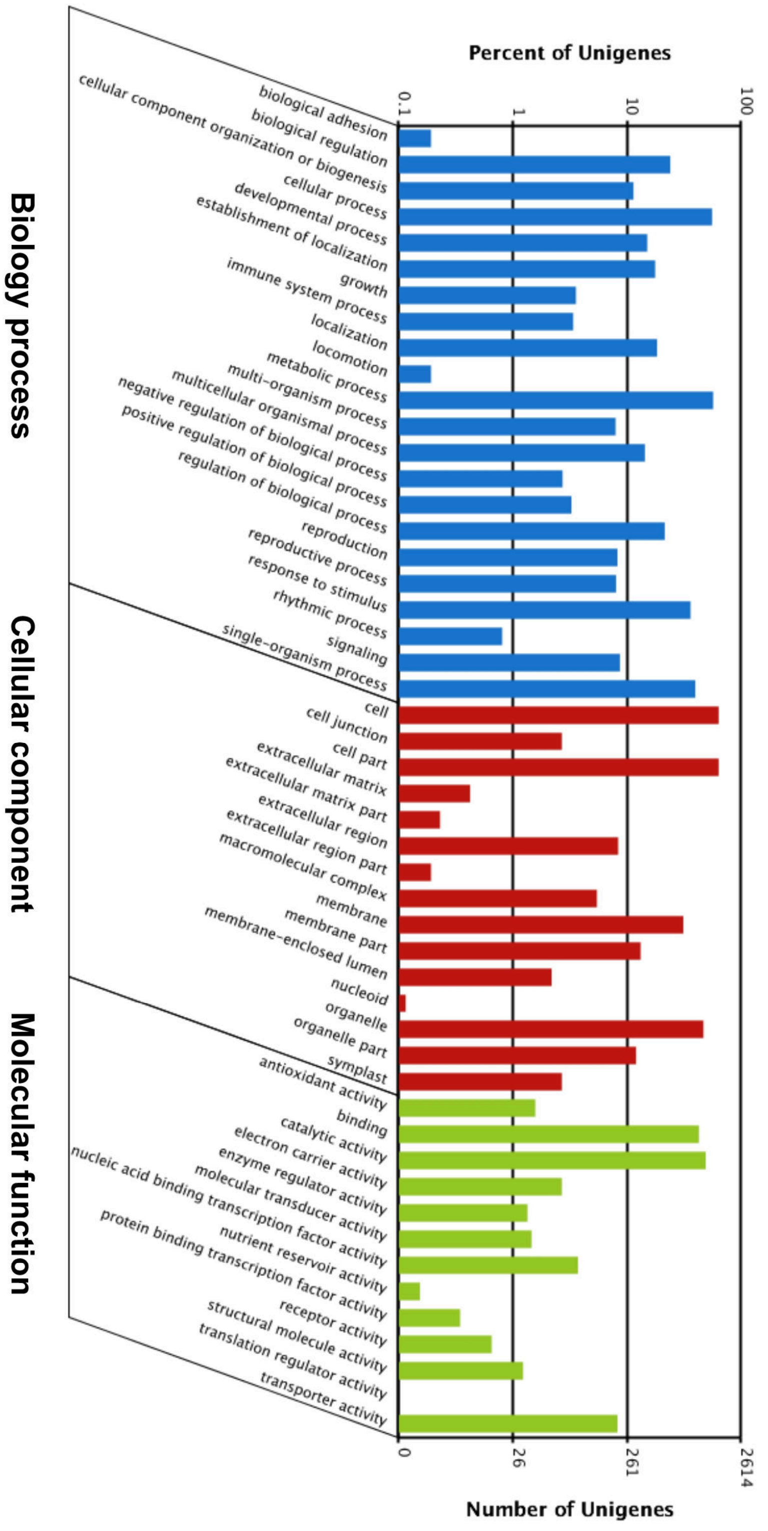

Figure S5. Classification of differentially expressed genes using GO.

GO functions was shown in X-axis. The left Y-axis shows the percentage of unigenes, the right Y-axis shows the number of genes which have the GO function.

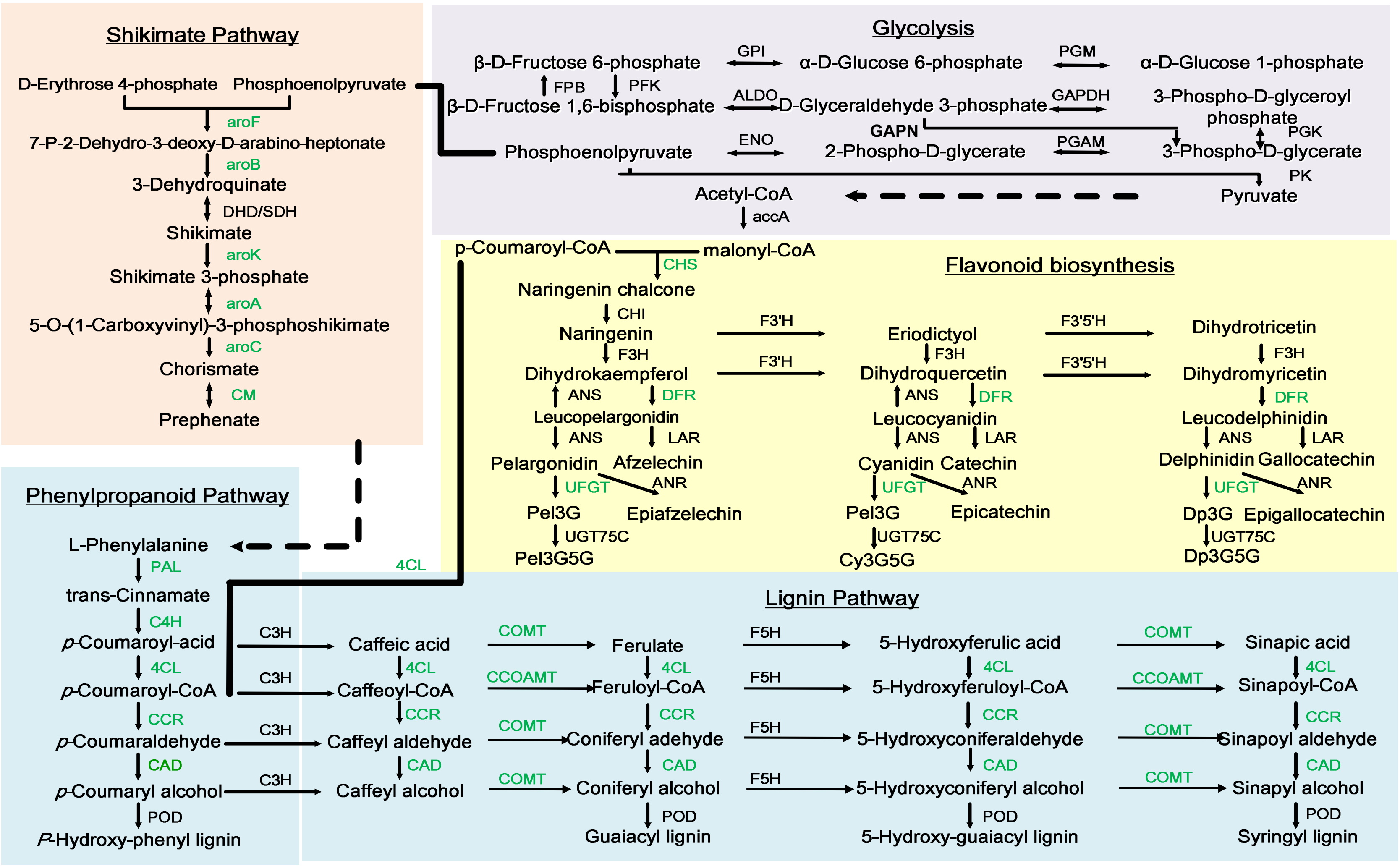

Shikimate Pathway
D-Erythrose 4-phosphate
Phosphoenolpyruvate
aroF
7-P-2-Dehydro-3-deoxy-D-arabino-heptonate
aroB
3-Dehydroquinate
DHD/SDH
Shikimate
aroK
Shikimate 3-phosphate
aroA
5-O-(1-Carboxyvinyl)-3-phosphoshikimate
aroC
Chorismate
CM
Prephenate
Glycolysis
β-D-Fructose 6-phosphate
GPI
α-D-Glucose 6-phosphate
PGM
α-D-Glucose 1-phosphate
FPB
PFK
β-D-Fructose 1,6-bisphosphate
ALDO
D-Glyceraldehyde 3-phosphate
GAPDH
3-Phospho-D-glyceroyl phosphate
GAPN
PGK
Phosphoenolpyruvate
ENO
2-Phospho-D-glycerate
PGAM
3-Phospho-D-glycerate
PK
Acetyl-CoA
Pyruvate
accA
p-Coumaroyl-CoA
malonyl-CoA
CHS
Flavonoid biosynthesis
Naringenin chalcone
CHI
Naringenin
F3'H
Eriodictyol
F3'5'H
Dihydrotricetin
F3H
Dihydrokaempferol
Dihydroquercetin
Dihydromyricetin
ANS
DFR
Leucopelargonidin
Leucocyanidin
Leucodelphinidin
LAR
Pelargonidin
Afzelechin
Cyanidin
Catechin
Delphinidin
Gallocatechin
UFGT
ANR
Pel3G
Epiafzelechin
Epicatechin
Dp3G
Epigallocatechin
UGT75C
Pel3G5G
Cy3G5G
Dp3G5G
Phenylpropanoid Pathway
L-Phenylalanine
PAL
trans-Cinnamate
C4H
p-Coumaroyl-acid
4CL
p-Coumaroyl-CoA
CCR
p-Coumaraldehyde
CAD
p-Coumaryl alcohol
POD
P-Hydroxy-phenyl lignin
Lignin Pathway
C3H
Caffeic acid
Caffeoyl-CoA
Caffeyl aldehyde
Caffeyl alcohol
COMT
CCOAMT
Ferulate
Feruloyl-CoA
Coniferyl adehyde
Coniferyl alcohol
Guaiacyl lignin
F5H
5-Hydroxyferulic acid
5-Hydroxyferuloyl-CoA
5-Hydroxyconiferaldehyde
5-Hydroxyconiferyl alcohol
5-Hydroxy-guaiacyl lignin
Sinapic acid
Sinapoyl-CoA
Sinapoyl aldehyde
Sinapyl alcohol
Syringyl lignin

Figure S6. Results from KEGG analysis show genes downregulated in transgenic plants by the overexpression of CsMYB4a.

Differentially expressed genes were submitted to KEGG to map their pathways and the RPKM value of each unigenes was used to compare its transcriptional level in transgenic and wild-type plants. Genes labeled with a greenish color indicates that their RPKM values were significantly lower in *CsMYB4a* transgenic tobacco as compared to those in wild-type plants.

Table S1. Oligonucleotide primers used in the experiments.

Primers	Sequence
	**Primer pairs used to clone CsMYB4a from tea leaves**
CsMYB4a orf F	ATGGGAAGGTCACCTTGCTG
CsMYB4a orf R	TCATTTCATCCCCAAGCTTCTGT
	**PCR program:** 98 ºC for 30s followed by 30 cycles of 98 ºC for 10s, 60 ºC for 20s, and 72 ºC for 20s, and then a 10min extension at 72 ºC
	**Primers used to Tobacco transformation**
Bp-F	AGGGGACAAGTTTGTACAAAAAAGCAGGCTATGGGAAGGTCACCTTGCTG
Bp-R	T GGGGACCACTTTGTACAAGAAAGCTGGGT CATTTCATCCCCAAGCTTCTGT
	**PCR program:** 98 ºC for 30s followed by 30 cycles of 98 ºC for 10s, 64 ºC for 20s, and 72 ºC for 20s, and then a 10min extension at 72 ºC
	**Primer pairs designed analysis of 20 tobacco gene expressions.**
Actain- F	TAGAAACCCCAAGTACCCTCG
Actain- R	TGCTTTCTTCGTCCCATCAG
UGT-F	TCTGGGCTCTAAGGAAACAACA
UGT-R	TTTCTTCCAAACATCGAGCATT
FLS-F	CTCCTTGCCCTATCCCTCATTT
FLS-R	TTCTCAGAGTTCACCACCACCC
DFR-F	CTGGTTGTTGGTCCATTCCTC
DFR-R	TGAATCTTCCCTCTGCCTTTG
CHS-Glike-F	CTGGTTGTTGGTCCATTCCTC
CHS-Glike-R	TGAATCTTCCCTCTGCCTTTG
COMT-F	TGATTGTCTCCAAACATCCCTC
COMT-R	ACTCCGCTATTATCACCTTCCC
CAD1-F	ACCAGAACAAGCAGCACCTCTA
CAD1-R	TGTCCCACTCCTCCTAATCCC
CCOAMT5-F	GCAAATAAAACAAACGAGAAAAGC
CCOAMT5-R	GGTAACCTGATTTTCCTGTGCTG
CCOAMT6-F	CAACATCAGCGGATGAAGGG
CCOAMT6-R	ATCAAGAACAGGCAAAGCAGG
CCR-F	TTCTTCCCGGAGTATCCTATCC
CCR-R	GTAAACTCCAGACCCAAATCCTT
4CL1-F	TGGCTACATTGATGATGACGAC
4CL1-R	CCACTGGAACTTCTCCTGCTT
4CL2-F	ACTGGCGACATTGGGTTCAT
4CL2-R	TTCTCCTGCTTGCTCGTCCT
C4H-F	TGGCGTTGGTAGGAGGAGTT
C4H-R	TCACAATGGTGGAATGCTTCA
PAL1-F	GGTGAGCCAAGTCGCAAAGA
PAL1-R	TGCAGGCGTCGTCAGCATAG
PAL2-F	TGTTCGCCTACGCTGATGAT
PAL2-R	CAATAGCAGGGTTGCCACAT
CM-F	TCCACCAGAGATGGACATCTGT
CM-R	GGAATTCATTGGAAAAGACCAG
aroC-F	GTGGCTTCGGAGGCACATT
aroC-R	AAGCGCCAGGGCTACCAT
aroA2-F	GTTAAGGAAACTGAGCGGATGA
aroA2-R	GTACTGCTGGAGAACGTCAAAGT
aroK-F	GTGGAGGTGCAGTTGTTCGTC
aroK-R	CTGGCACTTGCATTGGCATA
aroB-F	CCTATGCTATCAAGCGTTCCTG
aroB-R	AGTTTCTGGAGGTGAAGTTGGC
aroF-F	CAGGGCTGAAGTAAGAGCATTC
aroF-R	CCTCCTCTTTCTTAGGCGTTCT
	**PCR program:** 95 °C for 30 s, followed by 40 cycles at 95 °C for 5 s, and 60 °C for 30 s

	**Primer pairs designed to clone promoters of five tea phenylpropanoid genes**
proC4H-F	ATTTTCAATTGTACCAAATATC
proC4H-R	ATTGGCGTAGGTTAGAGGGAATAAT
pro4CL-F	TTAACAGCTATACCATTGAGTAGTGATAT
pro4CL-R	TGGGAATGAATTGGATGAAGAGAATGAAT
proCHS-R	TTGACCGACAAATTAAAAAAATTCACT
proCHS-R	TAGTAGTAGTAGAAACGTGAGCTTGT
proLAR-F	GACATATGATATTACCAATC
proLAR-R	TCTTTTCGCTACGTACCTTC
proANR2-F	AGTCCGGCCGGACTCCATGT
proANR2-R	CGGACAGTGGTGTTGACCGC
	**PCR program:** 98 ºC for 30s followed by 30 cycles of 98 ºC for 10s, 60 ºC for 20s, and 72 ºC for 30s, and then a 10min extension at 72 ºC
	Primers for dual-luciferase essays for tea promoters
C4H-F	GGGGACAAGTTTGTACAAAAAAGCAGGCT ATTTTCAATTGTACCAAATATC
C4H-R	GGGGACCACTTTGTACAAGAAAGCTGGGTATTGGCGTAGGTTAGAGGGAATAAT
4CL-F	GGGGACAAGTTTGTACAAAAAAGCAGGCT TTAACAGCTATACCATTGAGTAGTGATAT
4CL-R	GGGGACCACTTTGTACAAGAAAGCTGGGTTGGGAATGAATTGGATGAAGAGAATGAAT
CHS-R	GGGGACAAGTTTGTACAAAAAAGCAGGCT TTGACCGACAAATTAAAAAAATTCACT
CHS-R	GGGGACCACTTTGTACAAGAAAGCTGGGTTAGTAGTAGTAGAAACGTGAGCTTGT
LAR-F	GGGGACAAGTTTGTACAAAAAAGCAGGCT GACATATGATATTACCAATC
LAR-R	GGGGACCACTTTGTACAAGAAAGCTGGGTTCTTTTCGCTACGTACCTTC
ANR-F	GGGGACAAGTTTGTACAAAAAAGCAGGCT AGTCCGGCCGGACTCCATGT
ANR-R	GGGGACCACTTTGTACAAGAAAGCTGGGTCGGACAGTGGTGTTGACCGC
	**PCR program:**98 ºC for 30s followed by 30 cycles of 98 ºC for 10s, 60 ºC for 20s, and 72 ºC for 30s, and then a 10min extension at 72 ºC
	**Primers for dual-luciferase assays for tobacco promoters**
AROF-F	GGGGACAAGTTTGTACAAAAAAGCAGGCTTGAATGATTAGAAGAGAAAAGTTTAAAG
AROF-R	GGGGACCACTTTGTACAAGAAAGCTGGGTTGAATTTAATACTTTTATGAGAAA
AROC-F	GGGGACAAGTTTGTACAAAAAAGCAGGCT AGCATATTTTGCGATATTTAAAGG
AROC-R	GGGGACCACTTTGTACAAGAAAGCTGGGTTCGTCGTCGCTTTGACCTAC
	**PCR program:** 98 ºC for 30s followed by 30 cycles of 98 ºC for 10s, 60 ºC for 20s, and 72 ºC for 30s, and then a 10min extension at 72 ºC
	**Oligomeric nucleotide designed for development of probes used in EMSA**
AC-I-F	CGGTACCAGTCC**ACCTACC**GCC**ACCTACC**GCC**ACCTACC**GCTGTTCTCGA
AC-I-R	TCGAGAACAGCGGTAGGTGGCGGTAGGTGGCGGTAGGTGGACTGGTACCG
AC-II-F	CGGTACCAGTCC**ACCAACC**GCC**ACCAACC**GCC**ACCAACC**GCTGTTCTCGA
AC-II-R	TCGAGAACAGCGGTTGGTGGCGGTTGGTGGCGGTTGGTGGACTGGTACCG
AC-III-F	CCAGTCC**ACCTAAC**TCT**ACCTAAC**TCT**ACCTAAC**GCTGTTC
AC-III-R	GAACAGCGTTAGGTAGAGTTAGGTAGAGTTAGGTGGACTGG
AC-IV-F	CCAGTCC**ACCAAAC**TCT**ACCAAAC**TCT**ACCAAAC**GCTGTTC
AC-IV-R	GAACAGCGTTTGGTAGAGTTTGGTAGAGTTTGGTGGACTGG

The blue colored represent the forward AC-elements and the gray colored represent the reverse AC-elements.

Table S2. Pearson correlation coefficient analysis between CsMYB4a expression level and polyphenol compounds.

Compounds	Expression Level of *CsMYB4a*	
P-coumaroylquinic acid	Pearson Correlation	-0.907
	P Value	0.033*
Total Phenolic acids derivatives	Pearson Correlation	-0.893
	P Value	0.042*
Epicatechin gallate	Pearson Correlation	-0.893
	P Value	0.041*
Total Flavn-3-ols	Pearson Correlation	-0.830
	P Value	0.082

Table S3. **Statistic results from assembly of transcriptomic sequencing of *CsMYB4a* transgenic and wild-type tobacco plants.**

	Sample	Total Number	Total Length (nt)	Mean Length （nt）	N50	Total Consensus Sequences	Distinct Clusters	Distinct Singletons
Contig	WT	99937	28815580	288	443			
	MYB4a	105215	29591342	281	521			
Unigene	WT	56404	27481189	487	686	56404	21126	35278
	MYB4a	59078	27816724	471	658	59078	21432	37646
	All	52,290	32,069,182	613	778	52290	22957	29333

Table S4. Statistic results from annotation results of total unigenes sequenced from both *CsMYB4a* transgenic and wild-type tobacco plants.

Data base	NR	NT	Swiss-Port	KEGG	COG	GO
Unigene	33820	37795	19581	17056	9983	24881

Table S5. Results from functional characterizaiton via KEGG pathway enrichment analysis.

Pathway	Wt vs CsMYB4a (1853)	All-Unigene (17056)	Pvalue	Qvalue	Pathway ID	Level 2
Phenylpropanoid biosynthesis	99	364	1.600733e-18	1.984909e-16	ko00940	Biosynthesis of other secondary metabolites
Biosynthesis of secondary metabolites	369	2263	1.435441e-17	8.899734e-16	ko01110	Global map
Phenylalanine metabolism	57	185	1.084298e-13	4.481765e-12	ko00360	Amino acid metabolism
Plant hormone signal transduction	163	946	8.810783e-10	2.731343e-08	ko04075	Signal transduction
Diterpenoid biosynthesis	34	114	2.440866e-08	6.053348e-07	ko00904	Metabolism of terpenoids and polyketides
Flavonoid biosynthesis	41	158	7.571671e-08	1.564812e-06	ko00941	Biosynthesis of other secondary metabolites
Stilbenoid, diarylheptanoid and gingerol biosynthesis	47	195	1.013649e-07	1.795607e-06	ko00945	Biosynthesis of other secondary metabolites
Metabolic pathways	549	4234	3.546655e-07	5.497315e-06	ko01100	Global map
Fatty acid elongation	16	48	2.669855e-05	3.678467e-04	ko00062	Lipid metabolism
Other glycan degradation	26	107	5.955387e-05	7.384680e-04	ko00511	Glycan biosynthesis and metabolism
Zeatin biosynthesis	27	118	0.0001303084	1.383917e-03	ko00908	Metabolism of terpenoids and polyketides
Cutin, suberine and wax biosynthesis	21	82	0.0001339275	1.383917e-03	ko00073	Lipid metabolism
Isoflavonoid biosynthesis	18	67	0.0002069281	1.973776e-03	ko00943	Biosynthesis of other secondary metabolites
Anthocyanin biosynthesis	6	10	0.0002324581	2.058915e-03	ko00942	Biosynthesis of other secondary metabolites
Glutathione metabolism	31	152	0.0003954151	3.268765e-03	ko00480	Metabolism of other amino acids
(GPI)-anchor biosynthesis	26	121	0.0004914601	3.808816e-03	ko00563	Glycan biosynthesis and metabolism
Flavone and flavonol biosynthesis	21	96	0.001293351	9.433854e-03	ko00944	Biosynthesis of other secondary

						metabolites
Glycerophospholipid metabolism	50	300	0.001385608	9.545300e-03	ko00564	Lipid metabolism
Plant-pathogen interaction	98	681	0.002163447	1.411934e-02	ko04626	Environmental adaptation
Benzoxazinoid biosynthesis	12	46	0.002996306	1.857710e-02	ko00402	Biosynthesis of other secondary metabolites
Pentose and glucuronate interconversions	30	167	0.003885699	2.294413e-02	ko00040	Carbohydrate metabolism
Ether lipid metabolism	32	182	0.004119269	2.319665e-02	ko00565	Lipid metabolism
Carotenoid biosynthesis	29	161	0.004302604	2.319665e-02	ko00906	Metabolism of terpenoids and polyketides
Limonene and pinene degradation	25	136	0.005874112	3.034958e-02	ko00903	Metabolism of terpenoids and polyketides
Glucosinolate biosynthesis	8	28	0.008101764	4.018475e-02	ko00966	Biosynthesis of other secondary metabolites

Table S6. Accessions numbers of the proteins and genes which used in this paper.

Proteins sequences in phylogenetic tree analysis	ID
CsMYB4a	KY774676
VvMYB4a	NP_001268129.1
EgMYB1	CAE09058
AmMYB308	JQ0960
ZmMYB31	CAJ42202
ZmMYB42	CAJ42204
TaMYB4	AAT37167
PvMYB4a	JF299185
AtMYB4	AY519615
AtMYB7	AEC06531
AtMYB32	NP_195225
AtMYB3	NP_564176
AmMYB330	P81395
GhMYB9	AAK19619
GmMYB48	ABH02823
OsMYB1	BAA23337
HvMYB1	P20026

Genes involved in phenylpropanoid and shikimate pathways in tobacco plants	ID
NtAROF	NM_001325203.1
NtAROB(3-dehydroquinate synthase)	XM_009614286.2
NtAROK(tomentosiformis shikimate kinase)	XM_009627657.2
NtAROA2(3-phosphoshikimate 1-carboxyvinyltransferase)	XM_009782781.1
NtAROC(chorismate synthase)	XM_009784811.1
NtCM(chorismate mutase)	XM_009615669.2
NtPAL1(phenylalanine ammonia-lyase)	NM_001325423.1
NtPAL2(phenylalanine ammonia-lyase)	EU883670.1
NtC4H(cinnamic acid 4-hydroxylase)	NM_001325516.1
Nt4CL1(4-coumarate–CoA ligase)	NM_001325625.1
Nt4CL2(4-coumarate–CoA ligase)	NM_001325738.1
NtCCR(hybrida cinnamoyl-CoA reductase)	XM_016587861.1
NtCCOMT5(caffeoyl-CoA O-methyltransferase)	XM_016619959.1
NtCCOMT6(caffeoyl-CoA O-methyltransferase)	NM_001325865.1
NtCAD1(carbonic anhydrase)	NM_001325471.1
NtCOMT(caffeic acid 3-O-methyltransferase)	XM_016634241.1
Nt-CHSG-like(chalcone synthase)	XM_016660053.1
NtDFR(dihydroflavonol 4-reductase)	NM_001325732.1
NtFLS(flavonol synthase)	XM_016611945.1

UFGT(glucosyltransferase)	AB072919.1

Genes involved in phenylpropanoids pathway in tea plants	ID
CsC4H(cinnamic acid 4-hydroxylase)	KY615674
Cs4CL(4-coumarate–CoA ligase)	KY615679
CsCHS (chalcone synthase)	KY615680
CsLAR(leucoanthocyanidin reductase)	KY615697
CsANR2(anthocyanidin reductase)	KY615701
